# WEclMon – A simple and robust camera-based system to monitor *Drosophila* eclosion under optogenetic manipulation and natural conditions

**DOI:** 10.1371/journal.pone.0180238

**Published:** 2017-06-28

**Authors:** Franziska Ruf, Martin Fraunholz, Konrad Öchsner, Johann Kaderschabek, Christian Wegener

**Affiliations:** 1 Neurobiology and Genetics, Theodor-Boveri-Institute, Biocenter, University of Würzburg, Am Hubland, Würzburg, Germany; 2 Microbiology, Theodor-Boveri-Institute, Biocenter, University of Würzburg, Am Hubland, Würzburg, Germany; 3 Central workshop, Theodor-Boveri-Institute, Biocenter, University of Würzburg, Am Hubland, Würzburg, Germany; University of Lübeck, GERMANY

## Abstract

Eclosion in flies and other insects is a circadian-gated behaviour under control of a central and a peripheral clock. It is not influenced by the motivational state of an animal, and thus presents an ideal paradigm to study the relation and signalling pathways between central and peripheral clocks, and downstream peptidergic regulatory systems. Little is known, however, about eclosion rhythmicity under natural conditions, and research into this direction is hampered by the physically closed design of current eclosion monitoring systems.

We describe a novel open eclosion monitoring system (WEclMon) that allows the puparia to come into direct contact with light, temperature and humidity. We demonstrate that the system can be used both in the laboratory and outdoors, and shows a performance similar to commercial closed funnel-type monitors. Data analysis is semi-automated based on a macro toolset for the open imaging software Fiji. Due to its open design, the WEclMon is also well suited for optogenetic experiments. A small screen to identify putative neuroendocrine signals mediating time from the central clock to initiate eclosion showed that optogenetic activation of ETH-, EH and myosuppressin neurons can induce precocious eclosion. Genetic ablation of myosuppressin-expressing neurons did, however, not affect eclosion rhythmicity.

## Introduction

Many holometabolous insect species time their eclosion (i.e. the emergence of the adult insect from the pupa or adult ecdysis) not only to a specific time of season, but also to a specific time of day. This leads to overt daily eclosion rhythms on the population level, well known to entomologists and fly fishermen since centuries. Eclosion assays offer an ideal behavioural read-out to study molecular and cellular mechanisms of circadian timing, as eclosion timing continues under constant conditions and is basically unaffected by the masking influence of motivational or physiological states of the animal such as hunger, memory, age or reproductive state. Classic chronobiological experiments used eclosion assays with fruit flies (genus *Drosophila*) to demonstrate that behavioural rhythms can be influenced by abiotic factors such as light and temperature **[1–3]** and can be driven by an endogenous light-entrainable timing system **[1,3]**. A *Drosophila* eclosion assay was also used in the ground-breaking identification of *period* **[4]**, the first clock gene which was later on also found in vertebrates including humans **[5]**. For proper eclosion timing in *Drosophila*, both a central clock in the brain and a peripheral clock in the prothoracic gland are required **[6,7]**. Eclosion (ecdysis) behaviour itself is orchestrated by a peptidergic signalling cascade comprising hormonal cells as well as peptidergic neurons **[8,9]**. Results from *Drosophila* and moths indicate that the release of ecdysis-triggering hormone (ETH) from the epitracheal glands **[10–12]** initiates this cascade and activates subsets of down-stream peptidergic neurons in a stereotyped sequence **[13–16]**. How the clock in the brain and prothoracic gland times ETH release and the start of the ecdysis-orchestrating peptidergic cascade is, however, largely unknown.

Locomotor activity under laboratory conditions is another main behavioural read-out in circadian studies using *Drosophila* **[17]**. Recent experiments revealed, however, that *Drosophila* locomotor behaviour significantly changes in various aspects when flies are assayed under nature-like/seminatural conditions (e.g. **[18–20]**). At least part of these differences are likely due to the simultanous presence of different variable abiotic factors (e.g. light, temperature, humidity) that could act as Zeitgebers under natural conditions, while in the laboratory usually either light intensity or temperature is cycling. Moreover, in the laboratory, light intensity or temperature is typically varied in fixed rectangular cycles (e.g. lights on or off), while in nature, Zeitgebers show graded and much more complex patterns resulting in altered locomotor activity patterns (e.g. moonlight during the night, complex changes in wavelength composition and intensity of light during dusk and dawn **[21]**). For *Drosophila* eclosion, only two studies have so far been carried out under seminatural conditions with fruit flies placed outside in a shaded place **[22,23]**. Both studies revealed differences in eclosion timing and rhythmicity compared to laboratory conditions. However, in both studies, flies were enclosed in glass funnels or glass tubes and hence not freely exposed to temperature and humidity changes. Also the widely used “bang-box” funnel-type (see e.g. **[24,25]**, refined and commercialised by Trikinetics Inc. as *Drosophila* eclosion monitors) and “falling-ball”-type of eclosion monitors (see e.g. **[26–28]**), do not allow for an immediate and undampened exchange of temperature and humidity. In the funnel-type monitors, puparia are usually glued onto a plastic disc which is then put on top of a glass funnel. This closes the system with exception of the small funnel mouth that opens above a water-filled container. In the “falling-ball”-type of eclosion monitors, puparia are housed in wooden or plastic chambers which are closed by a movable plug. Light exposure may lead to increased temperature and subsequent humidity changes inside glass funnels or plastic chambers. Notwithstanding, buried falling-ball monitors have successfully been used under natural conditions to study eclosion timing of the onion fly (*Delia antiqua*) which pupariates 5–20 cm deep in the soil (**[29]**. Yet, already at these depths, light exposure is prevented, and temperature changes are dampened and occur at a much slower rate **[30]** compared to the soil/fruit surface or above-soil where many *Drosophila* species including *D*. *melanogaster* pupariate (see e.g. **[31–33]**, mitigating the draw-backs of the closed system.

To be able to address the significance of natural temporal fluctuations of abiotic factors for *Drosophila* eclosion, we developed an open monitoring system (Würzburg Eclosion monitor WEclMon) which allows an undampened direct contact of puparia and eclosing flies with outside changes in light, temperature and humidity and can be used both in the laboratory and in the field. The camera-based system is simple, robust, comparatively cheap and as reliable as existing commercial systems. In addition, it allows for a visual post-experiment correction of false positives/negatives and the making of behavioural videos. The WEclMon is also well suited for optogenetic experiments as shown by a small optogenetic screen to identify possible neuroendocrine signals that signal time from the central clock in the brain to the ETH-expressing peritracheal glands that are known to trigger ecdysis **[10,11]**.

## Material and methods

### Flies

Wildtype Canton S (CS) or Lindelbach were used for the outdoor experiments. *per*^*01*^ and *pdf*^*01*^ [34] flies were a kind gift of Charlotte Förster (U Würzburg, Germany). For optogenetic experiments, we relied on the *Gal4*-UAS-System [35] and drove expression of UAS-*ChR2-XXL* ([36], kind gift of Robert Kittel and Georg Nagel) flies by the following Gal4-lines: *Crz-Gal4* ([37], kind gift of Jan A Veenstra, U Bordeaux, France), *Dh31-Gal4* (Bloomington stock center, line# 51988 and 51989), *Dh44-Gal4* (Bloomington stock center, line# 39347, Vienna Drosophila Resource center line# VT039046), *Eh*-Gal4 ([38], kind gift of John Ewer, U Valparaiso, Chile), *ETH-Gal4* (kind gift of Michael Adams, UC Riverside, USA), *Hug-Gal4* ([39], kind gift of Michael Pankratz, U Bonn, Germany), *Ms-Gal4* ([40], kind gift of Jan A Veenstra), *Ptth-Gal4* ([41], kind gift of Naoki Yamanaka and Michael O’Connor, U Minnesota, USA), *sNPF-Gal4* ([42], kind gift of Dick Nässel, U Stockholm, Sweden), *Tdc2-Gal4* [43], *Th-Gal4*[44], and *Trh-Gal4* [45] (kind gifts of Dennis Pauls, U Würzburg, Germany). Flies were raised on *Drosophila* medium (0.8% agar, 2.2% sugarbeet syrup, 8.0% malt extract, 1.8% yeast, 1.0% soy flour, 8.0% corn flour and 0.3% hydroxybenzoic acid).

### Light and temperature entrainment experiments

For the experiments under light entrainment, flies were raised at 20°C with a light regime of 12 hours light and 12 hours darkness (LD 12:12) in the laboratory. After 12 to 17 days, puparia were collected and glued to the eclosion plate using a fungicide-free methyl cellulose glue (Tapetenkleister Nr. 389, Auro, Germany; 1:30 diluted in water). The plate with puparia was then mounted in the monitor and eclosion was monitored either under LD 12:12 or constant darkness (DD) at 20°C and 65% relative humidity. Infrared (λ_max_ = 850 nm) or red (λ_max_ = 635 nm) light was given throughout the experiment.

For the experiments under temperature entrainment, flies were raised at a temperature regime of 12 hours at 25°C and 12 hours at 16°C (WC 25:16). Red light (λ_max_ = 635 nm) was given constantly, from egg laying until the end of the experiment. During temperature shifts, temperature was increased and decreased by 1°C every 10 minutes. After 13 to 18 days, puparia were collected and glued to the eclosion plate, then placed in the monitors and eclosion was monitored either under WC 25:16 or a constant temperature at 20°C and 65% relative humidity.

### Experiments under natural conditions

Experiments under natural conditions were conducted from July to October 2014 in a little shelter shaded by bushes at the bee station/Hubland campus of the University of Würzburg. Flies were continuously bred outside in the shelter in large culture vials. Once most of the larvae had pupariated within a vial, it was transferred to the laboratory and puparia were collected and fixed on the eclosion plates as described above. Since flies were allowed to lay eggs for 3 to 4 days in each vial, the collected puparia were in different stages. On the same day, the eclosion plates with puparia were placed back into the shelter and eclosion was monitored for one week under constant red light (λ_max_ = 635 nm). In parallel we recorded light intensity, temperature and relative humidity using a datalogger (MSR 145S from MSR Electronics GmbH Seuzach, Switzerland) placed directly aside the monitors.

### Optogenetic experiments

For optogenetic activation, flies were raised inside of climate chambers (Percival Scientific, Inc., Plant Growth Chamber, DR-36NL, Perry, USA) under WC 25:16 temperature entrainment as described above. Retinal was not added to the food. After 14 to 19 days, pupae were collected and glued to the eclosion plate. The plates were then placed in open monitors inside the climate chamber and eclosion was monitored under WC 25:16 and 65% relative humidity. Red light (λ_max_ = 635 nm) was given constantly, from egg laying until the end of the experiment. Flies were glued onto the eclosion plate and monitoring was started at day 0. In the early night at day one (for *Ms-Gal4* experiments also on day 2–5), a 1h blue light pulse (λ_max_ = 470 nm, 586 μW/cm^2^) was given either 6 or 8h before the expected circadian eclosion peak.

### Data analysis

Rhythmicity index (RI) and period length of the eclosion profiles were analysed using autocorrelation and maximum entropy spectral analysis (MESA), respectively, implemented in an appropriate MATLAB (MathWorks, Inc., Natick, USA) toolbox [46]. Following standard conventions, an RI > 0.3 was defined as strongly rhythmic, an RI between > 0.1 and < 0.3 was defined as weakly rhythmic, and an RI < 0.1 was defined as arrhythmic. Statistical analysis with one-way ANOVA followed by Tukey post-hoc test and independent-samples t-test was performed using SPSS Statistics software (version 20). N depicts the number of independent experiments, n depicts the number of eclosed flies.

## Results

### Specifics of the *Würzburg Eclosion monitor* (WEclMon)

**Fig 1:**
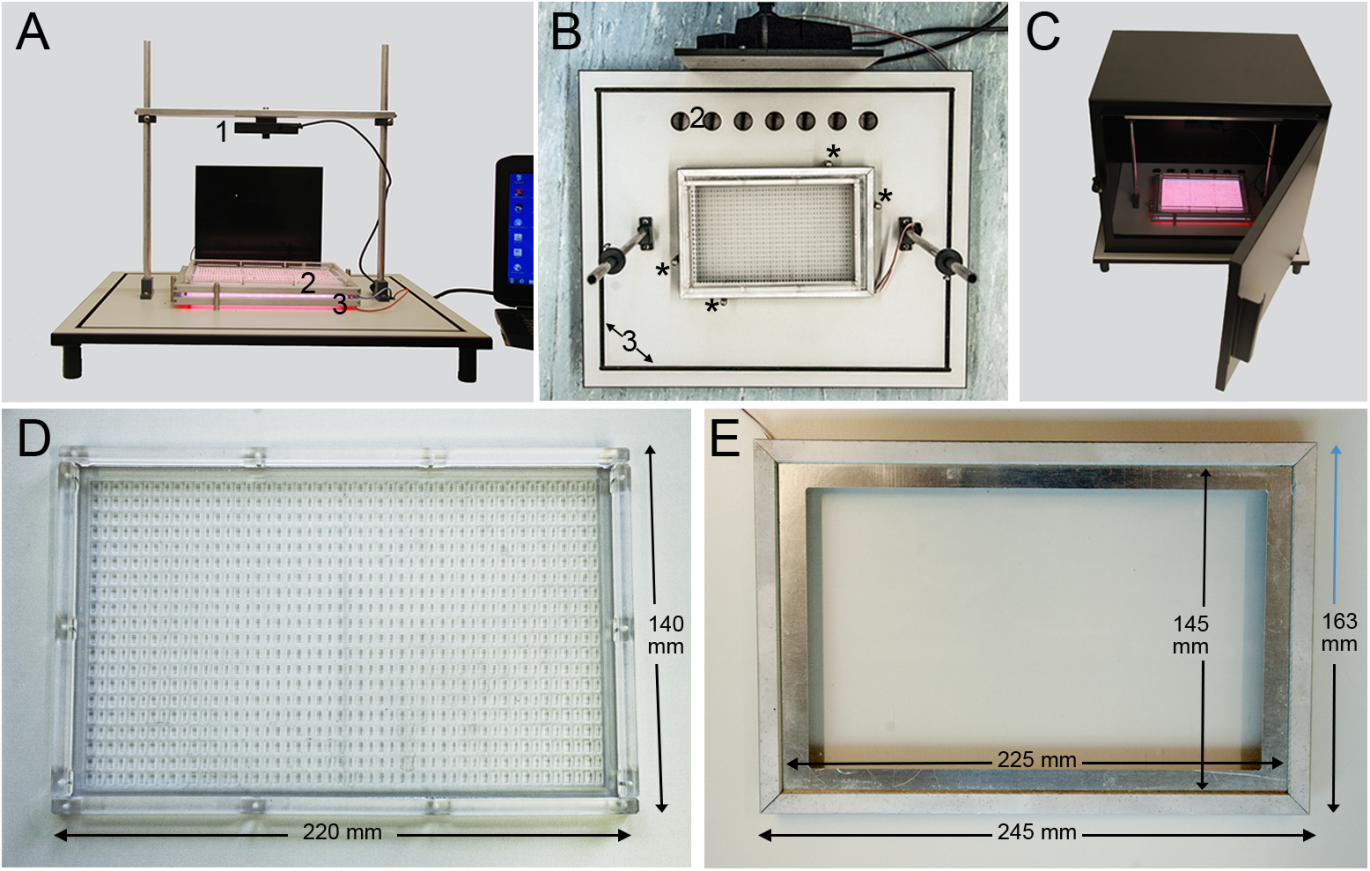
Setup of the WEclMon. **A, B)** The monitor consists of a webcam (1) mounted on a z-variable stage above an eclosion plate (2) on which the puparia are glued. Illumination for the camera and light regimes is provided by light plates stacked below the eclosion plate (3) and kept in place by four metal pins (asterisks in B),. The whole setup is mounted on a board, and is connected to a laptop computer. **B)** The monitor with removed camera seen from top. At the rear, a black plastic housing holds a multiplug connector with power supplies for the light plates (1). Holes in the laminate base (2) allow for humidity exchange and wiring via a light-trap when the system is closed by the light-tight box during DD experiments **(C)**. The light-tight box fits into a depression cut into the high-pressure laminate board (3 in B), and can be opened by a door which–when closed- is kept light-tight by a foam rubber lining. **D)** The eclosion plate with 1000 platforms. **E)** A light plate. The LED stripes surrounding the whole plate are covered by a metal frame and are thus not visible. The eclosion plate fits into the frame.

The WEclMon is an open monitoring system that allows experiments under natural abiotic conditions, such that puparia are directly exposed to the abiotic environmental factors, like temperature, light and humidity. Since mechanical stimuli may act as Zeitgebers [47], the WEclMon was designed as a camera-based system which is–unlike many funnel-type monitors- not subjected to mechanical agitation. The monitor consists of three main parts: a camera, light plates and an eclosion plate (Fig 1A). The eclosion plate (Fig 1D) is an acrylic glass plate containing 20 rows à 50 platforms, i.e. 1000 platforms/plate. Each platform is sized 4x2x2 mm, and separated by 2 mm from all neighbouring platforms. On each platform, a single puparium is placed and fixed with a drop of cellulose-based glue. On elevated platforms, flies had no difficulties to propel themselves out of the puparium, contrary to when we tried notches. Each acrylic eclosion plate is framed by a small metal band to reduce electrostatic charging. Even illumination for monitoring comes from below by lighting plates consisting of LED Stripes in red (12V SMD 3528 Red 60 LED/m; λ_max_ = 635 nm with a width from 595 to 655 nm, dimmable with max. 3.85W),) or infrared (YB-G3528IR60F08N12, IR850, 12V; λ_max_ = 850 nm, dimmable with max. 3.85W) fixed by a metal frame around an acrylic glass plate (Fig 1E) that can be stacked with the eclosion plate (Fig 1A–1C). For LD cycles, a light plate with white LEDs (12V SMD 3528 Cool White 60 Led/m; λ_max_ = 420–700 nm, dimmable with max. 3.85W) controlled by a standard commercially available clock timer is stacked with an infrared light plate. To activate ChR2-XXL, two arrays of blue LEDs (Luxeon V Star, LXHL-LB5C, λ = 470 nm, spectral half width 25 nm, luminous flux around 45 lm) are fixed on the left and right side of the camera stage.

As recording cameras, either a Logitech HD—C920 (Logitech, Romanel-sur-Morges, Switzerland, infrared (IR) filter manually removed) or a board camera Delock 95955 (Tragant Handels- und Beteiligungs GmbH, Berlin, Germany, built without IR filter) is used, but likely most other webcam types could be used too. To block day light, either a Chroma 625 /30 ET bandpass filter (AHF Analysentechnik AG, Tübingen, Germany) for the red LED light or an unexposed yet developed photographic film for infrared LED light is mounted in front of the camera. The monitoring camera is positioned centrally above the eclosion plate and connected to a notebook PC via a USB wire (Fig 1A). The camera can be moved up and down to either improve resolution or enlarge the field of view. This gives flexibility to either monitor eclosion of around 1000 individual flies at the same time or to monitor behavioural details in higher resolution in just a few flies. At the lowest position of our cameras that still produced focussed images, an array of 14x7 flies can be monitored at a resolution that allows to see ptilinum extension which happens shortly before eclosion [48] (S1 Movie).

All parts of the monitor are assembled onto a high-pressure laminate base ((Fig 1A and 1B). In this way, each monitor comes as a compact independent unit which can be directly placed into the field or in a climate chamber. For closed DD experiments in the laboratory, the whole setup can be covered by a light-tight metallic case (Fig 1C).

Intervalled image recording is controlled by the freeware Yawcam (http://www.yawcam.com). For image and data analysis, we developed a macro toolset for FiJi/ImageJ [49] which can be freely down-loaded at http://www.neurogenetics.biozentrum.uni-wuerzburg.de/services/wuerzburg_eclosion_monitor/. The macros exploit the fact that under transmitted light from the light plate below, the “dark” puparium with a pharate fly inside becomes translucent (“bright”) after eclosion. An eclosion event can therefore in principle be identified by an increase in light intensity. We found, however, that comparing the standard variation of light intensity between frames gives more stable results, most likely due to compensation of changes in the overall light intensities occurring over the course of a day.

The recorded PNG images are opened as image stack in FiJi, then converted to 8-bit gray scale and contrast-enhanced (saturated = 10). To minimize effects of different light conditions, the background is subtracted from each image using a rolling ball algorithm (diameter: 4, light background). Then, images are converted into binary images and puparia are identified by setting a threshold for intensity and subsequent particle analysis (size range 10 pixels to infinity). Square regions of interest (ROIs) with a side-length of 15 pixels are defined around the center of each particle and added to the ROI manager. These steps are automatically executed by the macro after threshold setting, yet ROIs should be visually checked for alignment and -if occurring- false-positive ROIs should be removed manually using FiJi’s ROI manager. A “MultiMeasure” macro then monitors the intensity of each ROI over the time course of the recorded images. A “detect_Eclosion” macro analyses the changes in intensity standard deviations between consecutive time points for each ROI. If a freely definable threshold of intensity/variation change between two images is crossed, this is registered as an eclosion event and the according ROI is highlighted. Two strategies are applied to prevent false-positive results: first, a ROI in which an eclosion event had been registered cannot be registered again. Second, a manual count of empty puparia on the eclosion plate after the experiment validates the number of calculated total hatches. A detailed protocol is given in S1 Text.

### WEclMon performance under standard light and temperature entrainment

**Fig 2:**
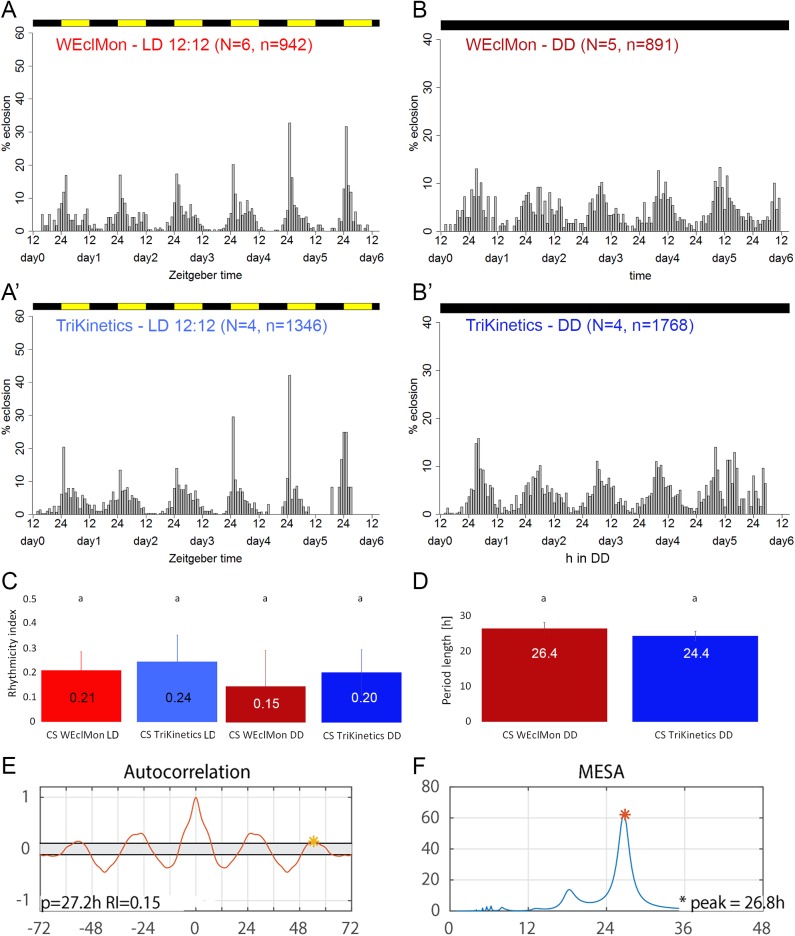
Comparison between eclosion rhythms in the WEclMon and TriKinetics monitor systems under laboratory conditions. Eclosion rhythms monitored by the WEclMon system equipped with infrared light source (**A, B**) and the TriKinetics system (**A’, B’**) under LD12:12 (A, A’), and DD (**B, B’),** after entrainment at LD12:12 at 20°C. Each bar represents the normalized percentage (number of flies eclosed in hour x/total number of flies eclosed during the day) of eclosion; colours represent the light regime. In both systems, lights-on eclosion peaks are visible under LD conditions (A, A’). **C)** Rhythmicity indices (RIs, ± s.d.) for the experiments shown in A-B’. In both systems and conditions, autocorrelation analysis indicated weak rhythmicity. The RI differences between monitors and conditions are not significant. **D)** Free-running periods (± s.d.) for the DD experiments shown in B and B’ are not significantly different between monitor types. **E)** Autocorrelation and **F)** MESA analysis results for eclosion rhythms monitored by the WEclMon system equipped with a red light source under DD. The RI of 0.15 and the period of 26.8h are similar to the values obtained with an infrared light source (see C-D), N = 5; n = 2056.

To test the WEclMon system, we first monitored eclosion rhythmicity of CS wildtype flies under laboratory conditions. Larvae were raised under a 12:12 hours light:dark (LD 12:12) cycle at 20°C and 65% relative humidity. Puparia were then mounted on eclosion plates and eclosion was monitored either under continued LD 12:12 cycle or under DD with constant infrared light for monitoring. The eclosion rhythms recorded were then compared with results from parallel experiments under the same LD and temperature conditions using the Trikinetics system (Fig 2). We could neither find significant differences in eclosion profiles (Fig 2A–2B’), nor in rhythmicity indices or period lengths between the WEclMon and the Trikinetics system (Fig 2C and 2D). We noted however, that the free-running period under DD in the WEclMon system was considerably longer (26.4h±1.8 s.d.) than the expected ~24h, though not statistically different from the 24.4h±1.4 free-running period measured using the Trikinetics system. We therefore repeated the measurements with the wildtype strain Lindelbach and obtained a period of 23.6±1.7h (WEclMon) and 25.0±0.4h (Trikinetics). This non-significant difference indicates that the continuous IR illumination under DD conditions in the WEclMon monitors are not affecting period length. Yet, we encountered an unexpected problem with IR illumination that required manual corrections: IR light reflected from eclosed flies that came very close a non-hatched puparium penetrated the puparium that in consequence became brighter. This lead to a light intensity increase which was often sufficient enough to be counted as a false positive by the eclosion macro.

Action spectra for eclosion entrainment for *D*. *melanogaster* and *D*. *pseudobscura* showed that light above 530 nm is not able to entrain eclosion rhythms [50,51]. This suggested that -alternative to IR light- also red light could be used as illumination source in the WEclMon. We therefore tested eclosion rhythmicity under DD in WEclMons equipped with a red light plate (λ_max_ = 635 nm). Under these conditions, we found that eclosion is rhythmic (RI = 0.15) with a free-running period of 26.8h in CS flies (Fig 2E and 2F), similar to the results obtained with infrared lighting (Fig 2C and 2D). Importantly, unlike IR, reflected red light did not penetrate the puparia and thus did not cause false positive eclosion calls. This suggests that red light >590 nm is better suited than IR to illuminate the puparia. Red light also allowed to check the functioning of the illumination and the status of puparia by eye even during darkness. Both can be advantageous for long-term and outdoor experiments.

**Fig 3:**
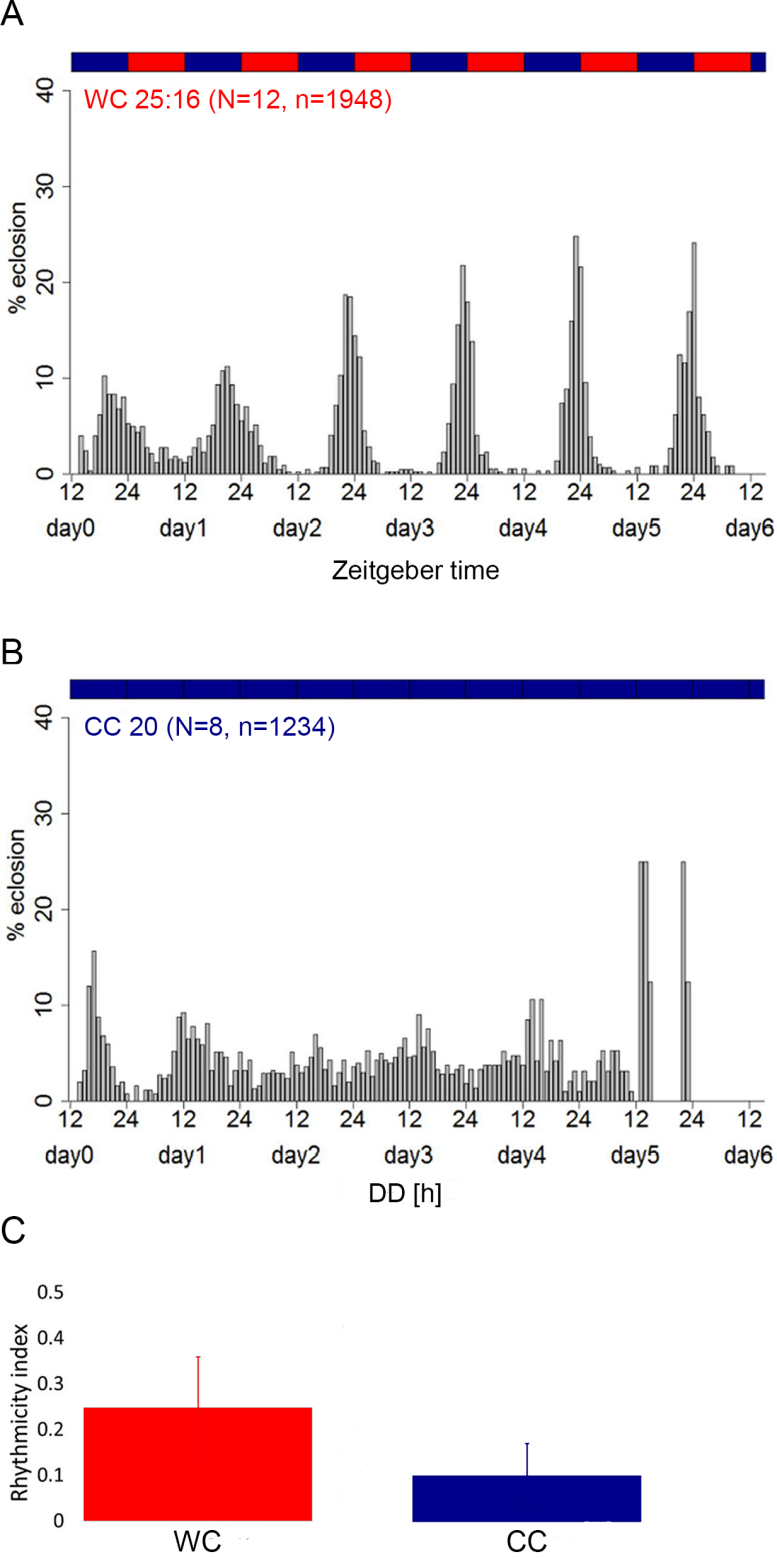
Eclosion rhythm after temperature entrainment in the WEclMon system. **A)** Eclosion rhythm of Canton S flies monitored by the WEclMon system equipped with a red light source under WC 25:16 entrainment and experimental condition. Eclosion is weakly rhythmic with a peak around onset of the warm period. **B)** Eclosion rhythm of Canton S flies at CC (constant 20°C) after entrainment at WC 25:16. Eclosion activity is weakly rhythmic with a peak at the end of the subjective cold period (Fig 2B and 2B’). **C)** The rhythmicity index (RIs, ± s.d.) under WC conditions is similar to that under LD conditions (Fig 2C), while under CC after WC entrainment, rhythmicity is considerably weaker then in DD after LD entrainment.

Next, we validated our setup for temperature entrainment experiments, using CS wildtype flies under a WC 25:16 temperature cycle or at constant 20°C (CC), 65% relative humidity and red light (λ = 635 nm, Fig 3). Under WC25:16, eclosion was weakly rhythmic (RI = 0.25±0.11), and remained so under CC though the rhythmicity index dropped as expected for temperature entrainment (Fig 3C).

Taken together, our results show that rhythmic eclosion can be monitored well with the WEclMon system under both light and temperature cycles in the laboratory. To test whether also arrhythmic eclosion in circadian gene mutants can be detected, we temperature-entrained *per*^*01*^ and *pdf*^*01*^ mutant flies as above and measured their eclosion rhythmicity under continued WC25:16 or under constant conditions (20°C). Both mutants had previously been shown to eclose arrhythmically under constant conditions [4,6,7]. In line, we observed arrhythmic eclosion of *per*^*01*^ in the WEclMon system (S1A and S1A” Fig). Consistent with earlier studies [6,7], *pdf*^*01*^ flies showed residual rhythmicity during the first 2 days under constant conditions, and then became arrhythmic (S1B Fig). In contrast, a continued WC25:16 cycle in the WEclMon system was sufficient to induce weak rhythmicity in both *per*^*01*^ and *pdf*^*01*^ mutants (S1A’–S1B” Fig).

### Experiments under natural conditions

**Fig 4:**
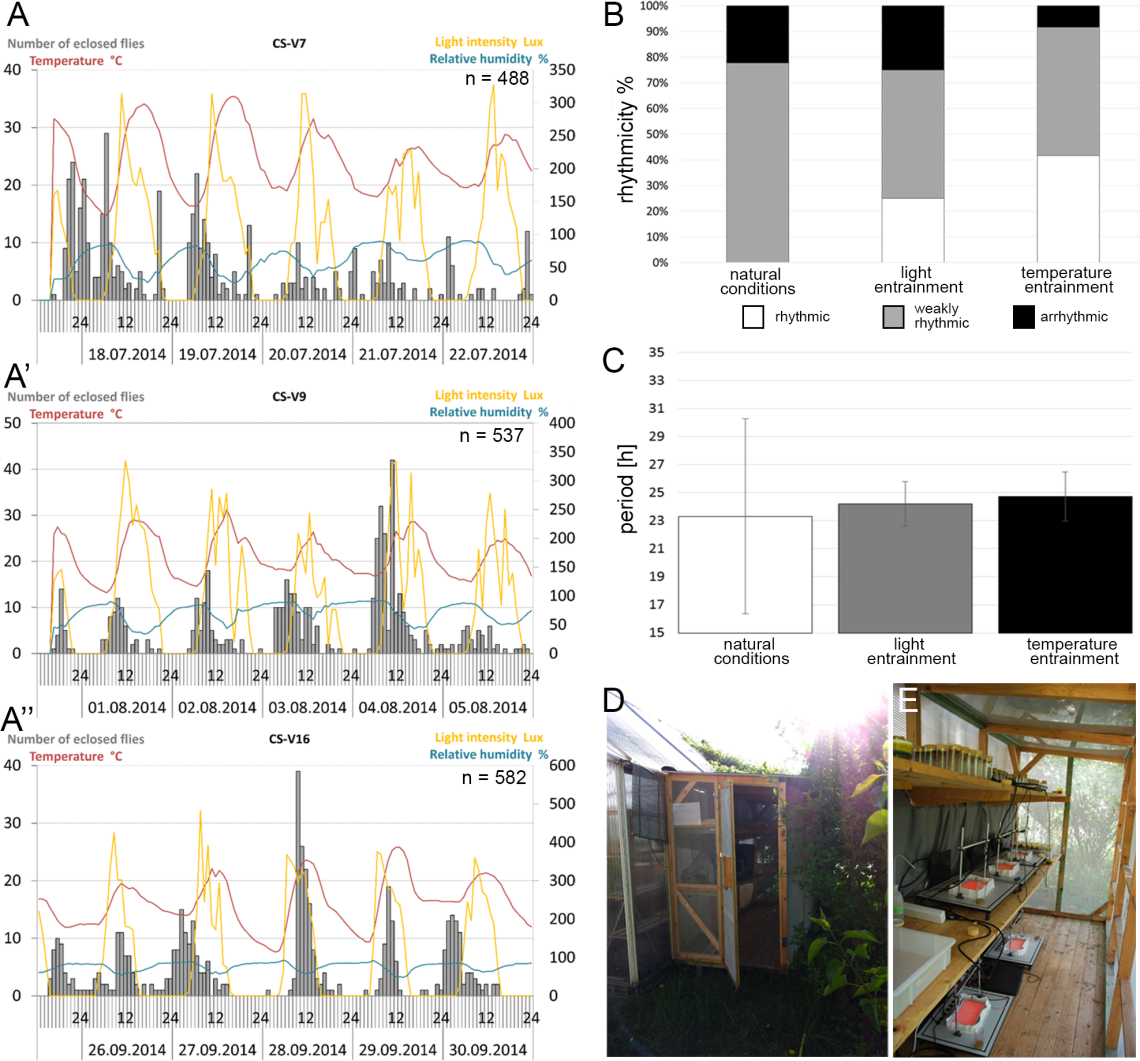
Outdoor eclosion. **A-A”)** Eclosion profiles (gray bars) and fluctuations in temperature (red curves), light intensity (yellow curves) and relative humidity (blue curves) from mid-July (**A**), beginning- of-August (**A’**), and end-of-September (**A”**). The temporal variability of eclosion within and between experiments is visible. **B-C)** Comparison of the eclosion rhythm strength **(B)** and period **(C)** of Canton S flies under natural outdoor conditions, and under light and temperature conditions in the laboratory. Rhythmicity is weakest and period is most variable under outdoor conditions. **D)** The outdoor shelter at the University’s bee station. Photograph taken in late afternoon with sun in the west. **E)** View inside the shelter. Flies were kept in normal vials on the top shelf. Monitors were placed in the lower shelf or on the floor.

To record eclosion rhythms under natural conditions, we placed WEclMons outside in a small shelter in a shaded area behind bushes and a glasshouse (Fig 4D and 4E). The shelter was roofed and in principle open from three sides. Yet, to keep predatory insects out and to prevent flies from escaping, the open sides had to be stretched with air-permeable black gauze. Additionally, double-sided sticky tape was glued around each monitor to trap predators and flies. Under outdoor conditions with the filters used, red light illumination was found superior to infrared illumination, since it resulted in more stable pixel intensity values over the course of a day. This facilitated image-analysis and reduced the rate of false positives and the need for manual post-hoc corrections.

Three examples for eclosion rhythms of wildtype CS flies under natural conditions are shown in Fig 4A–4A’”. S2 Movie contains a movie showing eclosion under natural conditions during one week recorded under red light illumination. The three experiments, conducted at different dates and temperatures, show quite different temporal eclosion profiles and reflect the high day-to-day temporal variance of eclosion under temperate natural conditions. In the first example during hot conditions reaching 35°C, eclosion was arrhythmic (Fig 4A). At temperatures staying within the pupation temperature range of *Drosophila* [52] (Fig 4A’ and 4A”) eclosion was weakly rhythmic. In the course of the first season in 2014, we performed 9 independent experiments. If all these results are taken together, 78% of the experiments showed a weak rhythm while 22% were arrhythmic (Fig 4B). Also the period length varied considerably, as seen by the high standard deviation (Fig 4C). Compared to laboratory experiments under light or temperature entrainment, eclosion rhythms under temperate natural conditions seem thus to be less robust with considerable day-to-day variations.

### Eclosion after optogenetic activation of peptidergic and aminergic cells

Optogenetics provide a powerful means to activate neurons in a precisely timed manner, but requires the possibility to directly shine light onto the experimental object. As the WEclMon is an open system, it should be well suited for optogenetic experiments. To test this, we monitored the timing of eclosion after optogenetic activation of a set of different peptidergic and aminergic neurons. As outlined in the introduction, eclosion is timed by clocks in the brain and prothoracic gland, and eclosion behaviour is orchestrated by a peptidergic cascade that can be started by release of ecdysis-triggering hormone (ETH) from the epitracheal glands [10,11]. As these glands are not innervated, we hypothesised that the signal timing eclosion might be a neuroendocrine hormone which, in a clock-controlled fashion, is released from the neurohemal organs of the brain, the corpora cardiaca. The peptide hormone complement of these organs is well characterised [53,54], and is supplied by different sets of peptidergic secretory neurons in the pars intercerebralis and pars lateralis of the protocerebrum [55,56]. Using specific Gal4 driver lines, we expressed the photoactivatable cation channel ChR2-XXL [36] in these different sets of neuroendocrine neurons, as well as in the epitracheal glands. We additionally expressed Chr2-XXL in various sets of aminergic neurons, since also biogenic amines can be hormonally released into in the hemolymph [57]. As proof-of-principle we first photoactivated the ETH-expressing epitracheal cells [11,12] under WC 25:16. As expected, a 1h blue light pulse given 6h ahead of the circadian eclosion peak triggered an immediate high rate of eclosion events, causing a precocious extra peak (S2 Fig). This effect was not seen when the light pulse was given 8h before the circadian eclosion peak (S2 Fig). It was also absent in genetic controls independent of the time of the blue light pulse (S2 Fig), showing that the blue light pulse itself is unable to induce eclosion.

**Fig 5:**
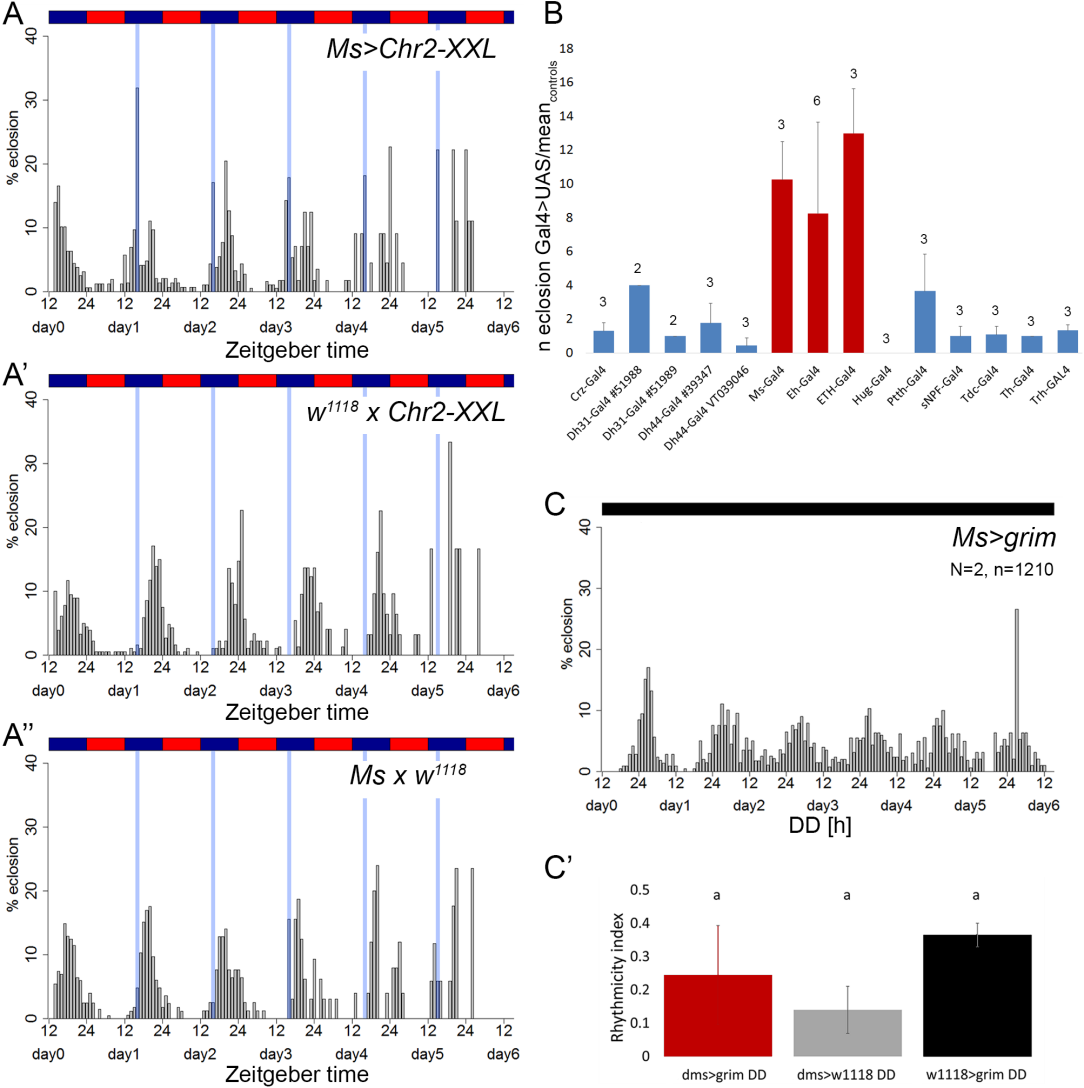
Eclosion profiles with optogenetic activation. **A-A”)** A 1h blue light pulse (indicated by a blue-shaded overlay) was given every early night 6h before the circadian eclosion peak in flies expressing Chr2-XXL in myosuppressing-expressing *Ms-Gal4* flies **(A)**, or UAS- **(A’)** and Gal4 –controls (**A”)**. A precocious eclosion peak could only be induced in flies with activatable *Ms-Gal4* neurons (A) Optogenetic activation via a blue light pulse had no effect in genetic controls (A’-A”). **B)** Mean ratio (± s.d.) between the number of eclosed flies of various peptide- or amine-*Gal4* cells expressing ChR2-XXL and the mean number of eclosed flies of the *Gal4* or UAS controls during blue light exposure. Only the activation of *Ms-Gal*, *Eh-Gal4* and *ETH-GAL4*-positive cells (red bars) led to increased eclosion compared to both controls. Numbers indicate the number of experiments (N). **C, C’)** Eclosion profile of flies with genetically ablated *Ms-Gal4* cells (**C**) shows the same weak rhythmicity as the genetic controls (**C’,** N = 2,2; n = 741, 1666).

Optogenetic activation of most of the other peptidergic and all aminergic lines tested did not result in precocious eclosion (Fig 5B, S3 Fig). Interestingly, however, activation of either *Ms*-*Gal4* (Fig 5A–5A”) or *Eh-Gal4* (S3 Fig) neurons triggered immediate eclosions. This provides first evidence that besides the expected ETH and EH, also myosuppressin or a co-localised factor may act as eclosion-triggering hormone in *Drosophila*. We did not observe an overt eclosion phenotype or reduced viability in flies when *Ms-Gal4* neurons were ablated by ectopical expression of the preapoptotic gene *grim* (*Ms>grim*). Ablation of myosuppressin neurons also did not impair eclosion rhythmicity (Fig 5C and 5C’).

## Discussion

Our results show that the new WEclMon system is suitable to analyse *Drosophila* eclosion under different entrainment regimes as well as under natural abiotic conditions. Under standard light entrainment in the laboratory, WEclMons showed a similar performance as the widely used Trikinetics eclosion monitors which suggests that the mechanical agitation in the funnel-type Trikinetics monitors is not influencing eclosion rhythmicity. Moreover, as the WEclMon is a physically open system, puparia are immediately and directly exposed to all abiotic changes in the environment, and are easily accessible for optogenetic manipulations. The monitors come for a reasonable price since they are largely made of non-expensive commercially or freely available components and software. As the system is camera-based, there was no data loss, for example by blocking of funnels or by “clever flies” like *D*. *littoralis* [28] that are able to stay some time inside the funnel. By comparing the empty puparia on the plate to the outcome from the image analysis, it was possible to check for and largely exclude false positive results.

Using the WEclMon system, eclosion rhythmicity under natural abiotic conditions was found to be weaker and sometimes even arrhythmic compared to laboratory conditions. This seems to be in contrast to the results of De et al. [23] who reported a more robust eclosion rhythm under seminatural conditions than under laboratory conditions. An explanation for this discrepancy may be that De and colleagues performed their experiments in Bangalore, India (12° 57′ N) where the temperature conditions are much more stable (seasonal and day-to-day temperature changes are smaller) than under the temperate conditions of Würzburg (49° 48′ N). Since De and colleagues kept their flies in tubes, humidity and temperature changes may also have been smoothened compared to the WEclMon conditions. It seems thus possible that day-to-day variation in the temperature or humidity profile induces variability in the timing of eclosion, leading to a lower autocorrelative rhythmicity index within an experiment typically running over 4–5 days.

Our small optogenetic screen shows that the WEclMon system is well suited for optogenetic manipulations during pupal and pharate development. We were able to induce precocious and immediate eclosion by optogenetic activation of the ETH-expressing epitracheal cells 6 hours prior to the circadian eclosion peak. Activation of the epitracheal cells two hours earlier was ineffective in inducing eclosion. This finding is in line with a six-hour eclosion gate [58], and underlines the importance of ETH for triggering eclosion behavior [10,12]. Photoreceptor-dependent activation of EH neurons underlies the well-characterised lights-on eclosion peak in *Drosophila* [59], and EH injections into the hemolymph triggers precocious ecdysis [60,61] and ETH release [62,63] in moths. The strong eclosion-triggering effect of eclosion hormone in *Drosophila* is thus little astonishing and emphasises also for adult eclosion the essential role of EH recently shown for larval ecdysis in *Drosophila* [64]. Yet, we were surprised to find that activation of *Ms-Gal4* cells triggered precocious ecdysis, as myosuppressin neurons had so far not been implicated in the regulation of *Drosophila* ecdysis, and myosuppressin has not been considered to be a part of the peptidergic cascade orchestrating ecdysis [8,9]. However, there is some evidence for a function of *Manduca sexta* myosuppressin (F10: pE**DV**V**H**S**FLRFa** (amino acids identical to *Drosophila* MS highlighted in bold)) and related FLRFamides during ecdysis based on its developmental and (neuro)endocrine expression profile [65]. Detailed mechanistic studies on myosuppressin action during ecdysis are, however, lacking for moths. Clearly, further detailed studies are also required for *Drosophila* to define the possible role and significance of myosuppressin neurons during eclosion. Based on our results, we can, at least, rule out a non-redundant role of myosuppressin for eclosion timing. Together with the lack of effect upon activation of other peptidergic and aminergic neuroendocrine cells, our results thus suggest EH neurons and–if redundant to EH action- also *Ms-Gal4* neurons as good candidates to mediate a time signal from the central clock to the peptidergic cascade regulating eclosion behaviour.

## Supporting information

S1 Movie Eclosion movie (1 frame/5 min) at higher resolution (camera close to eclosion plate). Ptilinium extension and eclosion movements can be observed.
(AVI)

S2 Movie Movie showing a full experiment with Canton S wildtype under natural conditions. The WEclMon was equipped with red light illumination.
(AVI)

S1 Text Manual for data analysis using Fiji and the eclosion toolset. (PDF)

S1 Fig Eclosion profiles of *per*^*01*^ (**A-A’**) and *pdf*^*01*^ (**B-B’**) mutant flies. Flies were temperature-entrained under WC25:16, and then monitored in the WEclMon system either under maintained WC25:16 (**A, B**) or under constant 20°C (**A’, B’**). **A”**) and **B”**): mean rhythmicity indices (± s.d.), N = 6, 6; n = 995, 1146 for *per*^*01*^ flies, N = 8, 12; n = 735, 948 for *pdf*^*01*^ flies.
(PDF)

S2 Fig Eclosion profiles with optogenetic activation of *ETH*-*Gal4* neurons 6h and 8h before the circadian eclosion peak. (PDF)

S3 Fig Eclosion profiles with optogenetic activation of peptide/amine-*Gal4* neurons 6h before the circadian eclosion peak. (PDF)
