# Supplementary material for: WEclMon – A simple and robust camera-based system to monitor Drosophila eclosion under optogenetic manipulation and natural conditions
Source: PLoS One. 2017 Jun 28;12(6):e0180238. doi: 10.1371/journal.pone.0180238 (PMC5489222; doi:10.1371/journal.pone.0180238)
Supplement: S1 Text — (PDF) [file pone.0180238.s003.pdf]

WEclMon toolset- Eclosion detection Macros (Ruf et al., PLOS ONE),

Macro developed by Martin Fraunholz and Franziska Ruf

Wegener lab, Neurobiology and Genetics, Uni Würzburg

Short instructions, V001

Download the WEclMon toolset (WEclMon-Tools.ijm) from our webpage:

http://www.neurogenetics.biozentrum.uni-wuerzburg.de/services/wuerzburg_eclosion_monitor/

Then, save the WEclMon tool set in your ImageJ/macros/toolsets folder. The toolset will appear in the >> menu at the right end of the ImageJ toolbar.

Select the toolset from the >> menu and the tools contained in it will be installed the tool bar. Four new buttons should appear now (labeled A-D in the figure below. A = Enhance button, B = Measure button, C = Call eclosion button, D = Adjust measure variables).

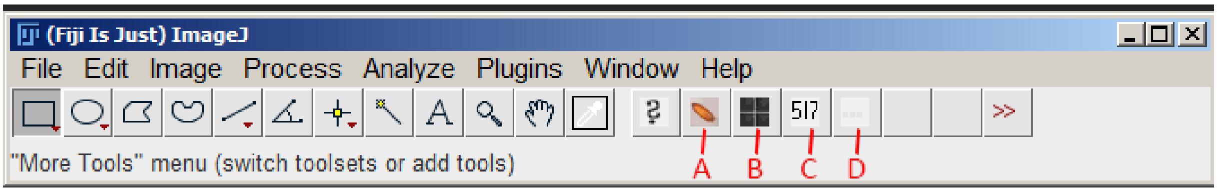

If you want to restore the default tool set, select "Startup Macos" from the >> menu.

1. Drag the folder of your Image series on Fiji (not necessary to convert to RGB) and open as a stack → an image stack will be created

2. use rectangular selection tool, select area to be measured. Then right click > duplicate > duplicate as stack → a new image stack containing the region of interest will be created.

3. Clicking on the “Enhance button” 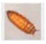 will convert the image to 8 bit grayscale, subtract background and enhance the contrast for the whole stack (will take a minute depending on stack size and resolution). Follow the instructions, and change the threshold if necessary.
 → ROIs are stored in the ROI Manager. Erroneously called ROIs can be deleted or additional ROIs can be added using the ROI Manager.

4. Run the multiMeasure tool by clicking on the “multiMeasure button” 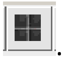.
→ This will measure intensities and standard deviations for all ROIs and frames

5. Run the detect_Eclosion tool by clicking on the “call eclosion button” 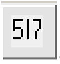.
→This will analyse eclosions per frame.

To change the sensitivity or ROI size, click on the “adjust measure variables button” 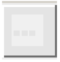. Ticking “verbose mode” will provide a more detailed output. You can also choose whether a simple cumulative graph is plotted.

6. → In the log window you will find the number of detected events and if you hide the ROIs in the ROI Manager by unchecking “Show All”, you will find the ROIs highlighted in magenta for which Eclosion has been detected. ROIs where no Eclosion has been detected are coloured yellow.

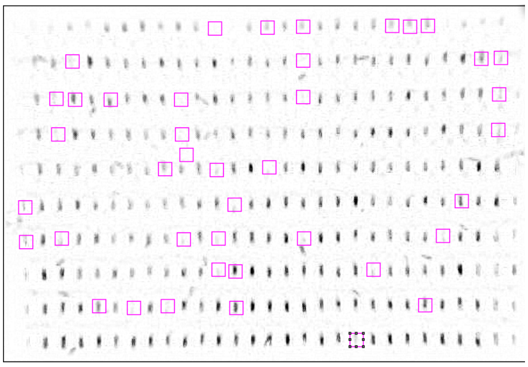

7. Count the number of eclosed flies on your eclosion plate, and compare with the calculated number of eclosed flies. If the numbers don’t match, run step 5 again with changed sensitivity. To double-check the results, compare the pattern of the ROIs with detected eclosion with the pattern of eclosed flies on the original eclosion plate.

For suggestions and questions, please contact us: christian.wegener@biozentrum.uni-wuerzburg.de
