# Supplementary figures and images for: WEclMon – A simple and robust camera-based system to monitor Drosophila eclosion under optogenetic manipulation and natural conditions
Source: PLoS One. 2017 Jun 28;12(6):e0180238. doi: 10.1371/journal.pone.0180238 (PMC5489222; doi:10.1371/journal.pone.0180238)

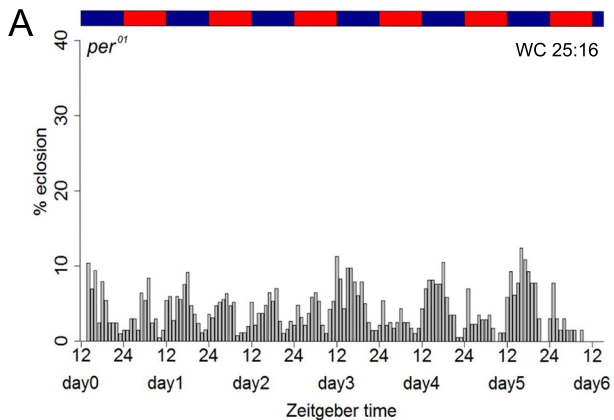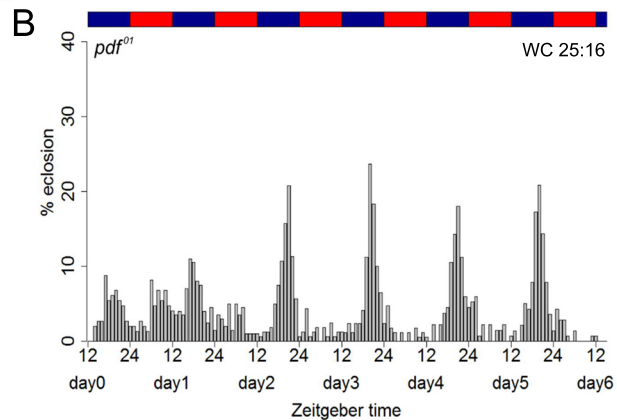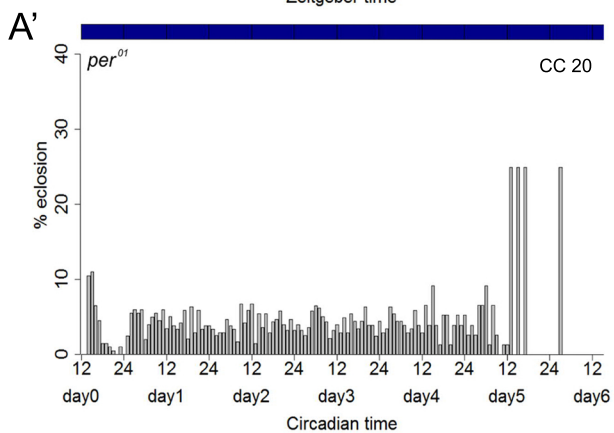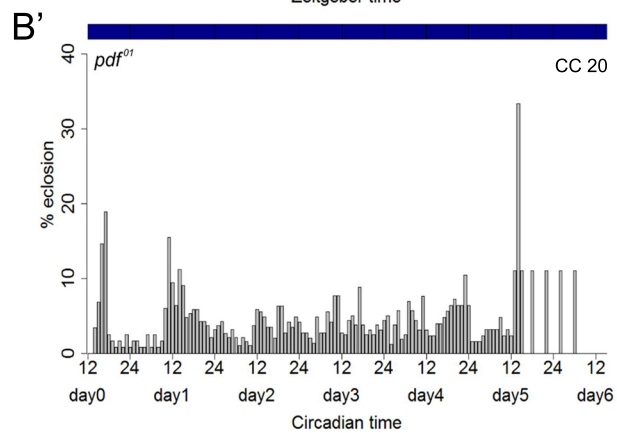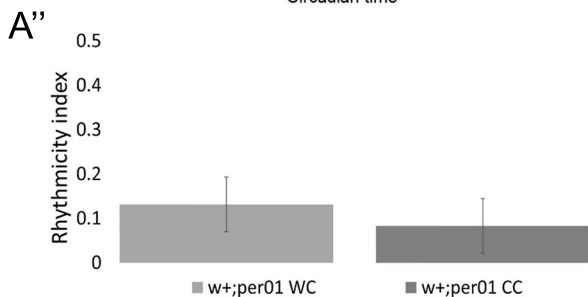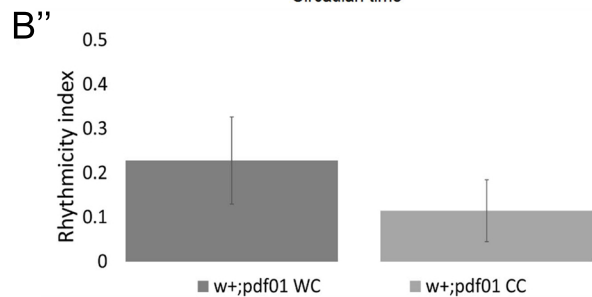

Supplement: S1 Fig — Eclosion profiles of per01 (A-A’) and pdf01 (B-B’) mutant flies. Flies were temperature-entrained under WC25:16, and then monitored in the WEclMon system either under maintained WC25:16 (A, B) or under constant 20°C (A’, B’). A”) and B”): mean rhythmicity indices (± s.d.), N = 6, 6; n = 995, 1146 for per01 flies, N = 8, 12; n = 735, 948 for pdf01 flies. (PDF) [file pone.0180238.s004.pdf]
