# Supplementary material for: WEclMon – A simple and robust camera-based system to monitor Drosophila eclosion under optogenetic manipulation and natural conditions
Source: PLoS One. 2017 Jun 28;12(6):e0180238. doi: 10.1371/journal.pone.0180238 (PMC5489222; doi:10.1371/journal.pone.0180238)
Supplement: S3 Fig — (PDF) [file pone.0180238.s006.pdf]

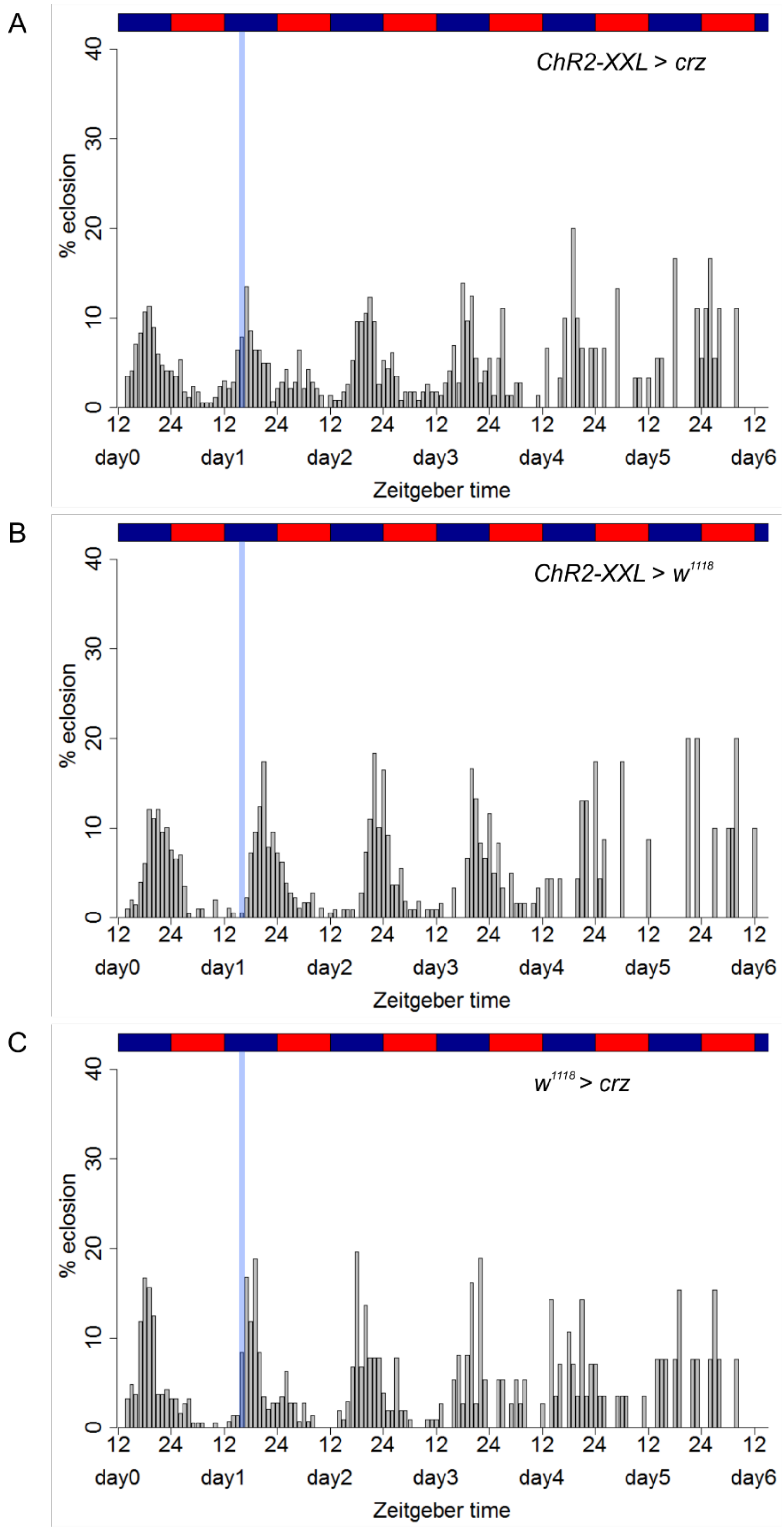

Eclosion profiles with optogenetic activation of *Crz-Gal4* neurons 6h before the circadian eclosion peak

Eclosion profiles for populations expressing ChR2-XXL driven by *crz*-GAL4 (A) and the respective controls (B: UAS control, C: GAL4 control) under temperature entrainment (25°C:16°C). Each bar represents the percentage of eclosed flies per hour normalized to the number of eclosed flies per day. The blue and red rectangles represent the temperature regime. The blue bars mark the time point of activation with blue light. (N=3, 3, 3; n=544, 580, 511)

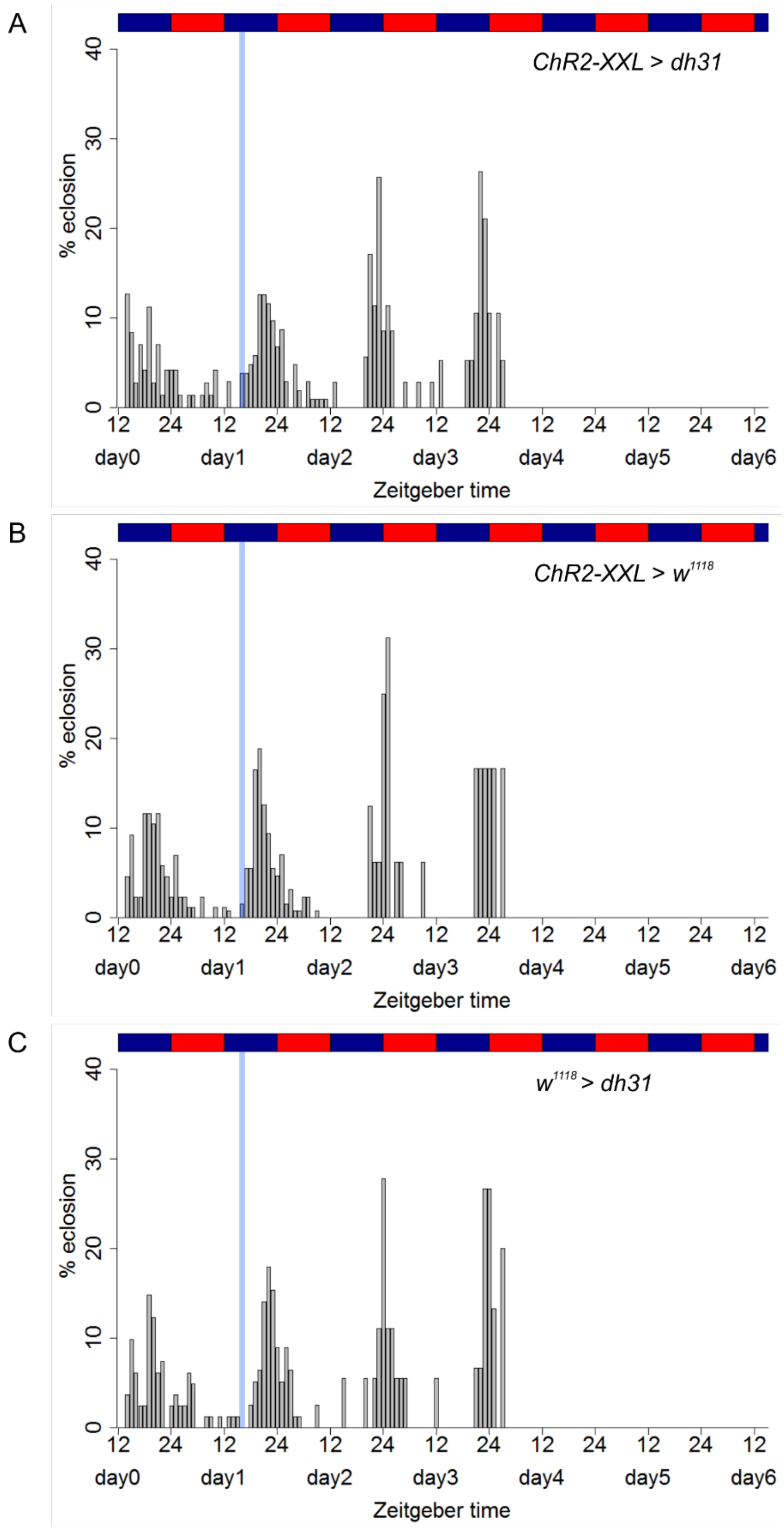

Eclosion profiles with optogenetic activation of D*h31*-*Gal4* neurons 6h before the circadian eclosion peak

Eclosion profiles for populations expressing ChR2-XXL driven by *Dh31*-GAL4 (#51988) (A) and the respective controls (B: UAS control, C: GAL4 control) under temperature entrainment (25°C:16°C). Each bar represents the percentage of eclosed flies per hour normalized to the number of eclosed flies per day. The blue and red rectangles represent the temperature regime. The blue bars mark the time point of activation with blue light. (N=2, 2, 2; n=228, 235, 192)

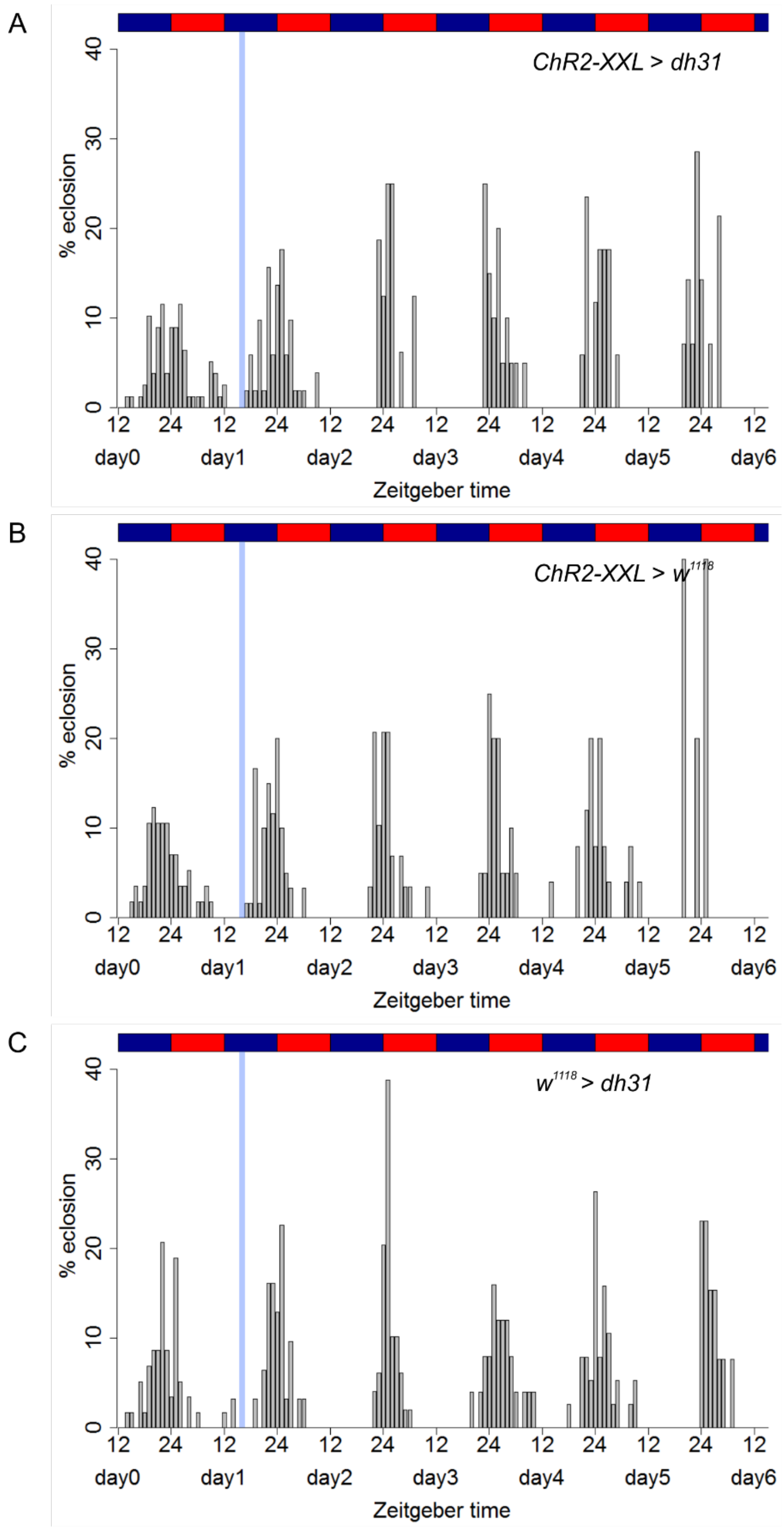

Eclosion profiles with optogenetic activation of *Dh31-Gal4* neurons 6h before the circadian eclosion peak

Eclosion profiles for populations expressing ChR2-XXL driven by *Dh31*-GAL4 (#51989) (A) and the respective controls (B: UAS control, C: GAL4 control) under temperature entrainment (25°C:16°C). Each bar represents the percentage of eclosed flies per hour normalized to the number of eclosed flies per day. The blue and red rectangles represent the temperature regime. The blue bars mark the time point of activation with blue light. (N=2, 2, 2; n=196, 196, 214)

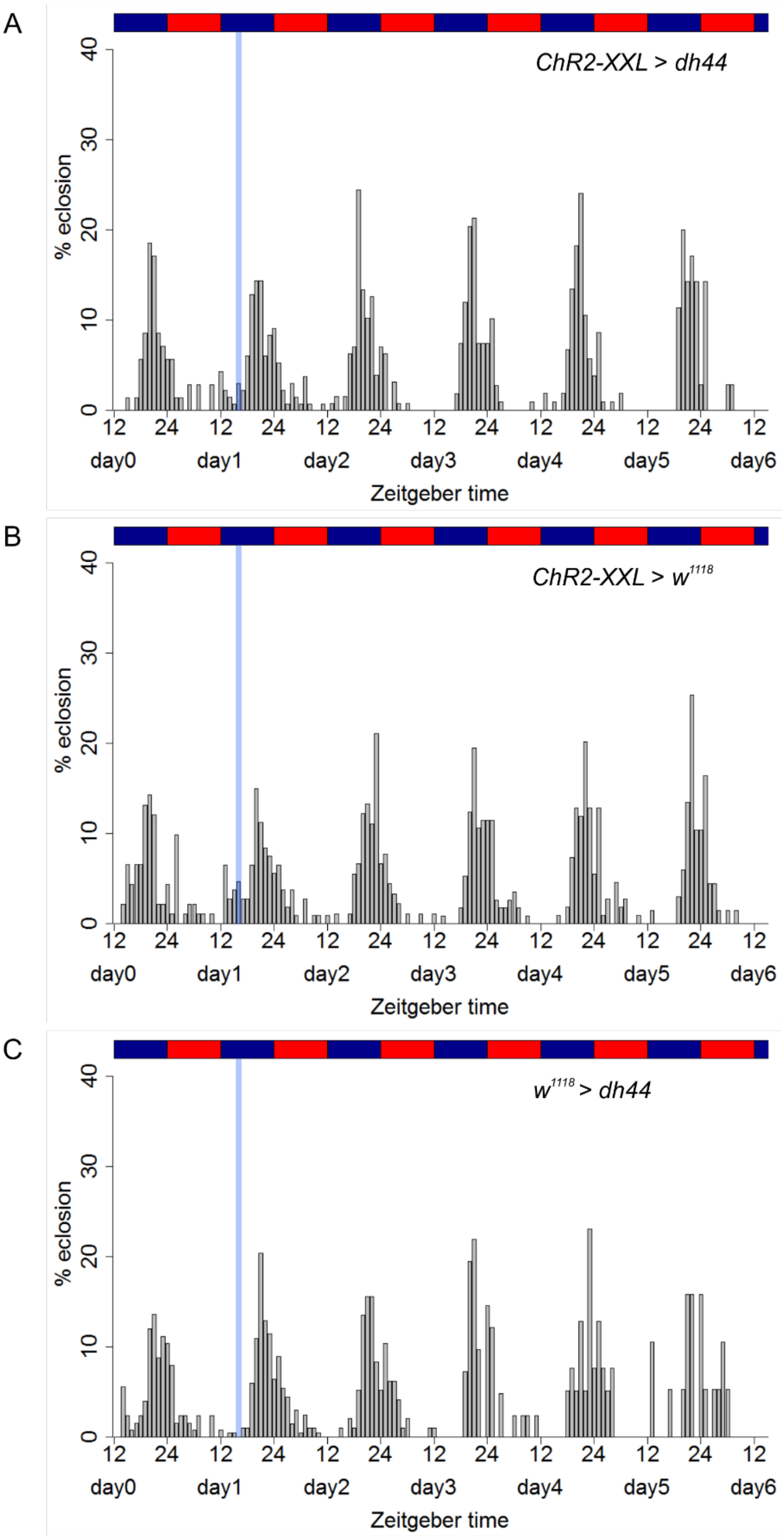

Eclosion profiles with optogenetic activation of *Dh44-Gal4* neurons 6h before the circadian eclosion peak

Eclosion profiles for populations expressing ChR2-XXL driven by *Dh44*-GAL4 (#39347) (A) and the respective controls (B: UAS control, C: GAL4 control) under temperature entrainment (25°C:16°C). Each bar represents the percentage of eclosed flies per hour normalized to the number of eclosed flies per day. The blue and red rectangles represent the temperature regime. The blue bars mark the time point of activation with blue light. (N=3, 3, 3; n=576, 577, 521)

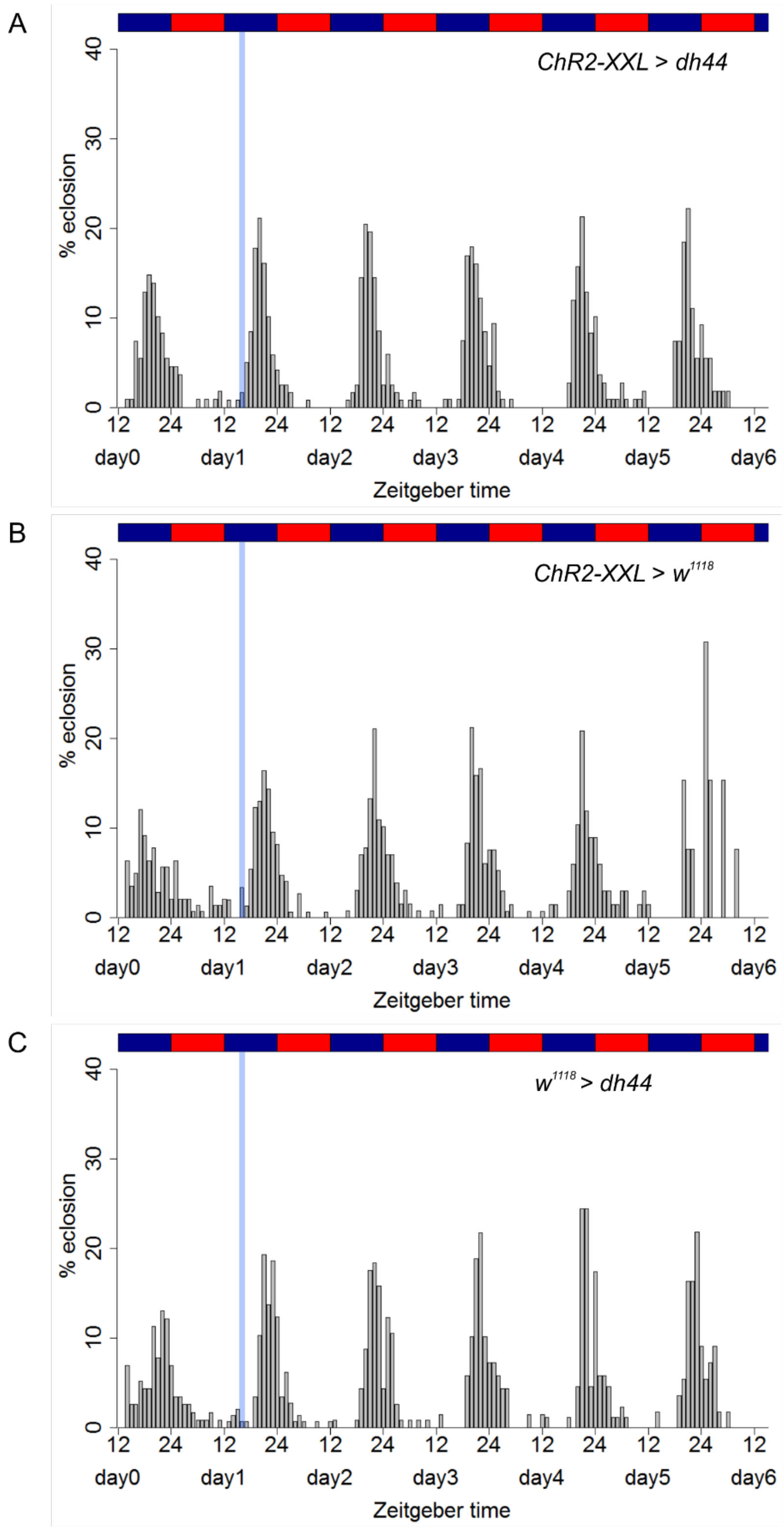

Eclosion profiles with optogenetic activation of *Dh44-Gal4* neurons 6h before the circadian eclosion peak

Eclosion profiles for populations expressing ChR2-XXL driven by *Dh44*-GAL4 (VT039046) (A) and the respective controls (B: UAS control, C: GAL4 control) under temperature entrainment (25°C:16°C). Each bar represents the percentage of eclosed flies per hour normalized to the number of eclosed flies per day. The blue and red rectangles represent the temperature regime. The blue bars mark the time point of activation with blue light. (N=3, 3, 3; n=611, 627, 584)

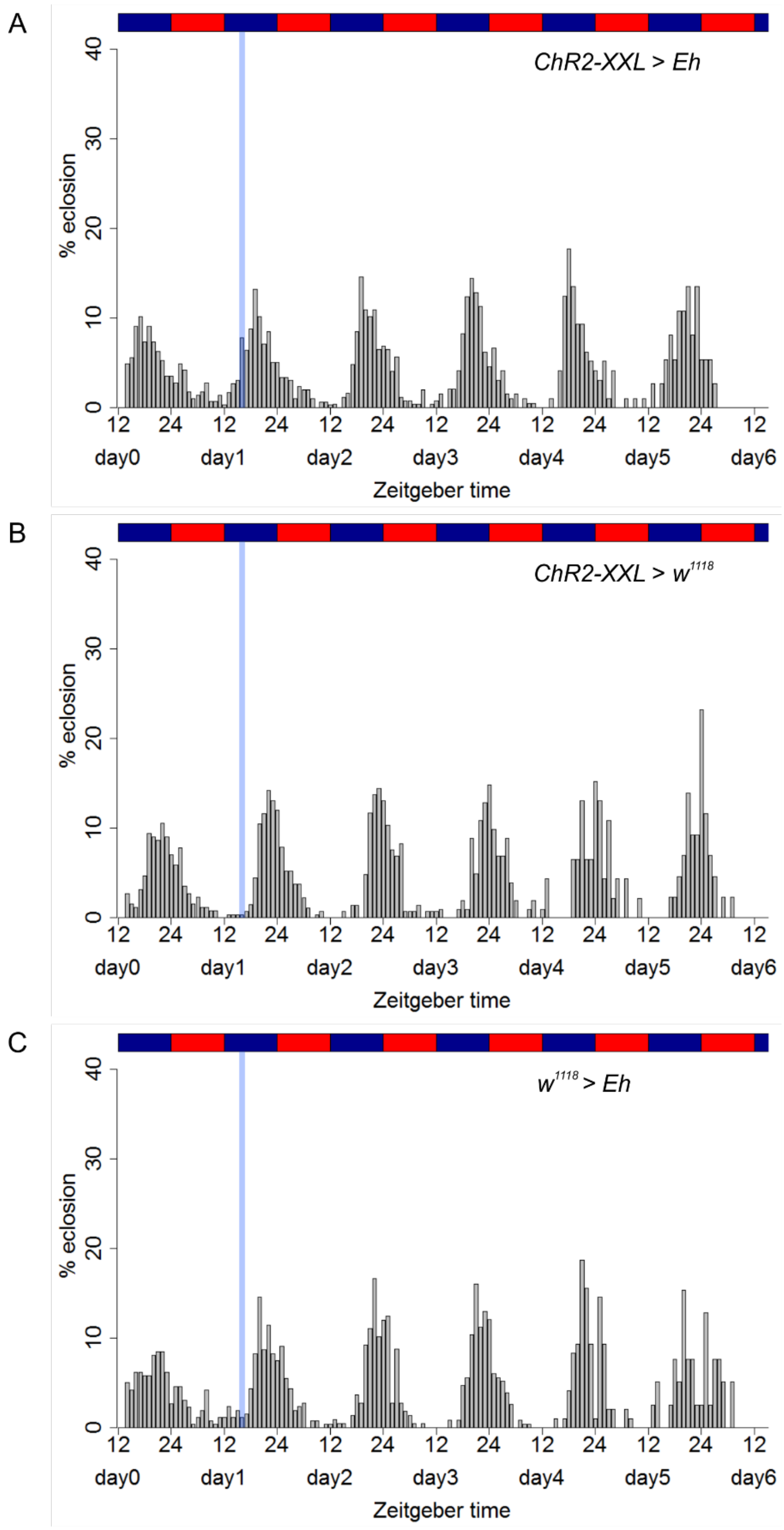

Eclosion profiles with optogenetic activation of *Eh-Gal4* neurons 6h before the circadian eclosion peak

Eclosion profiles for populations expressing ChR2-XXL driven by *Eh*-GAL4 (A) and the respective controls (B: UAS control, C: GAL4 control) under temperature entrainment (25°C:16°C). Each bar represents the percentage of eclosed flies per hour normalized to the number of eclosed flies per day. The blue and red rectangles represent the temperature regime. The blue bars mark the time points of activation with blue light. (N=6, 6, 6; 1152, 857, 1093)

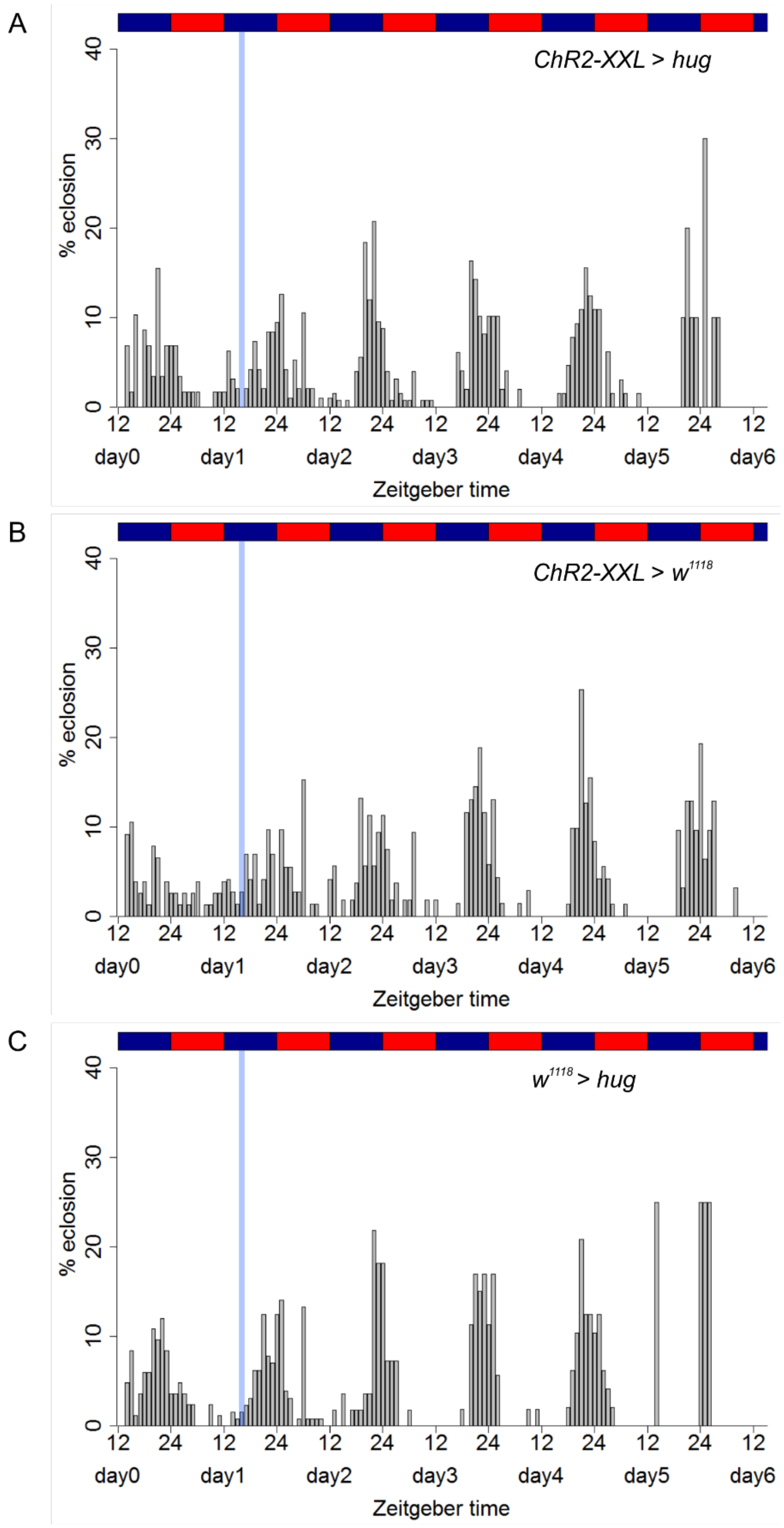

Eclosion profiles with optogenetic activation of *Hug-Gal4* neurons 6h before the circadian eclosion peak

Eclosion profiles for populations expressing ChR2-XXL driven by *Hug*-GAL4 (A) and the respective controls (B: UAS control, C: GAL4 control) under temperature entrainment (25°C:16°C). Each bar represents the percentage of eclosed flies per hour normalized to the number of eclosed flies per day. The blue and red rectangles represent the temperature regime. The blue bars mark the time points of activation with blue light. (N=3, 3, 3; n=401, 372, 371)

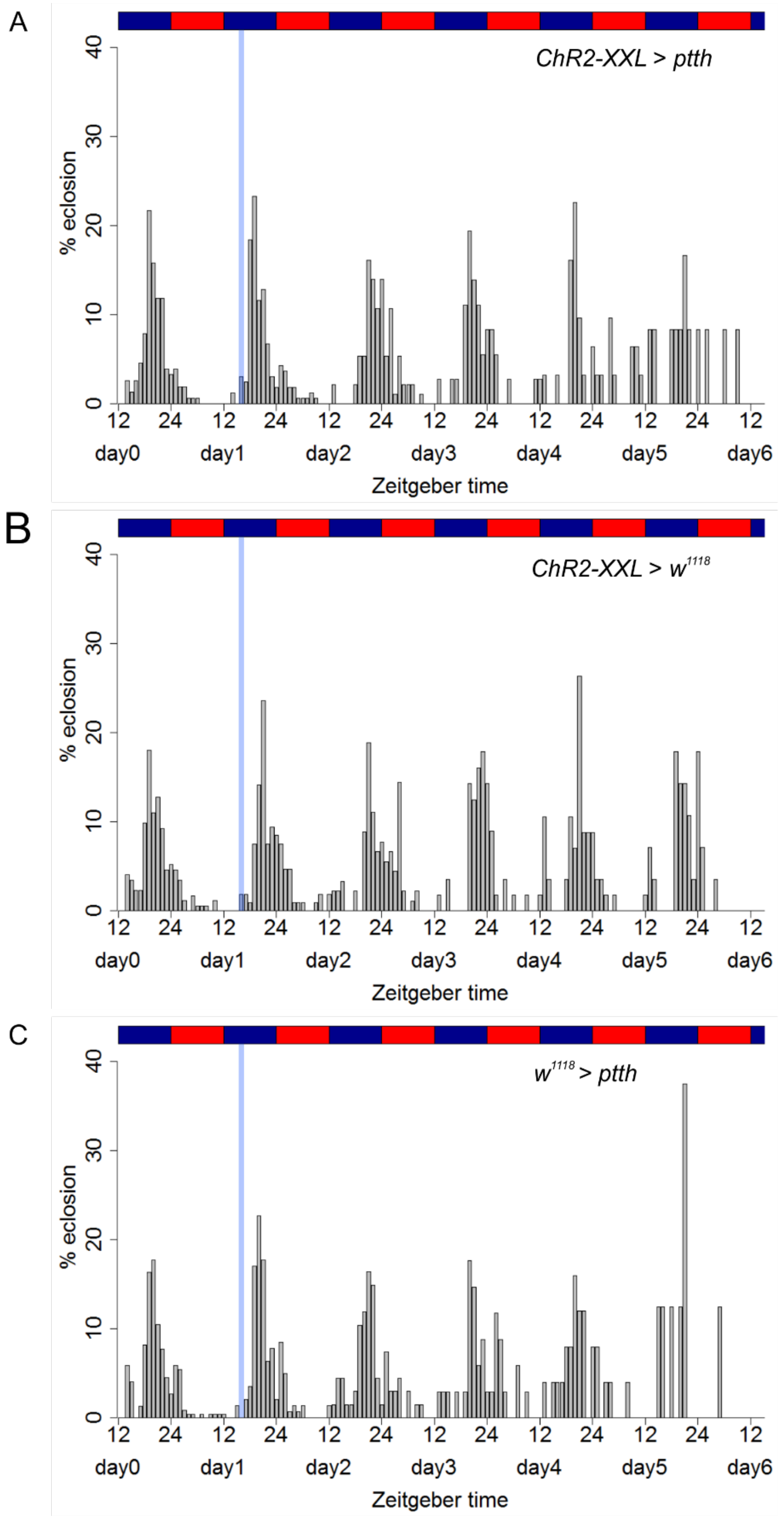

Eclosion profiles with optogenetic activation of *Ptth-Gal4* neurons 6h before the circadian eclosion peak

Eclosion profiles for populations expressing ChR2-XXL driven by *Ptth*-GAL4 (A) and the respective controls (B: UAS control, C: GAL4 control) under temperature entrainment (25°C:16°C). Each bar represents the percentage of eclosed flies per hour normalized to the number of eclosed flies per day. The blue and red rectangles represent the temperature regime. The blue bars mark the time points of activation with blue light. (N=3, 3, 3; n=488, 510, 495)

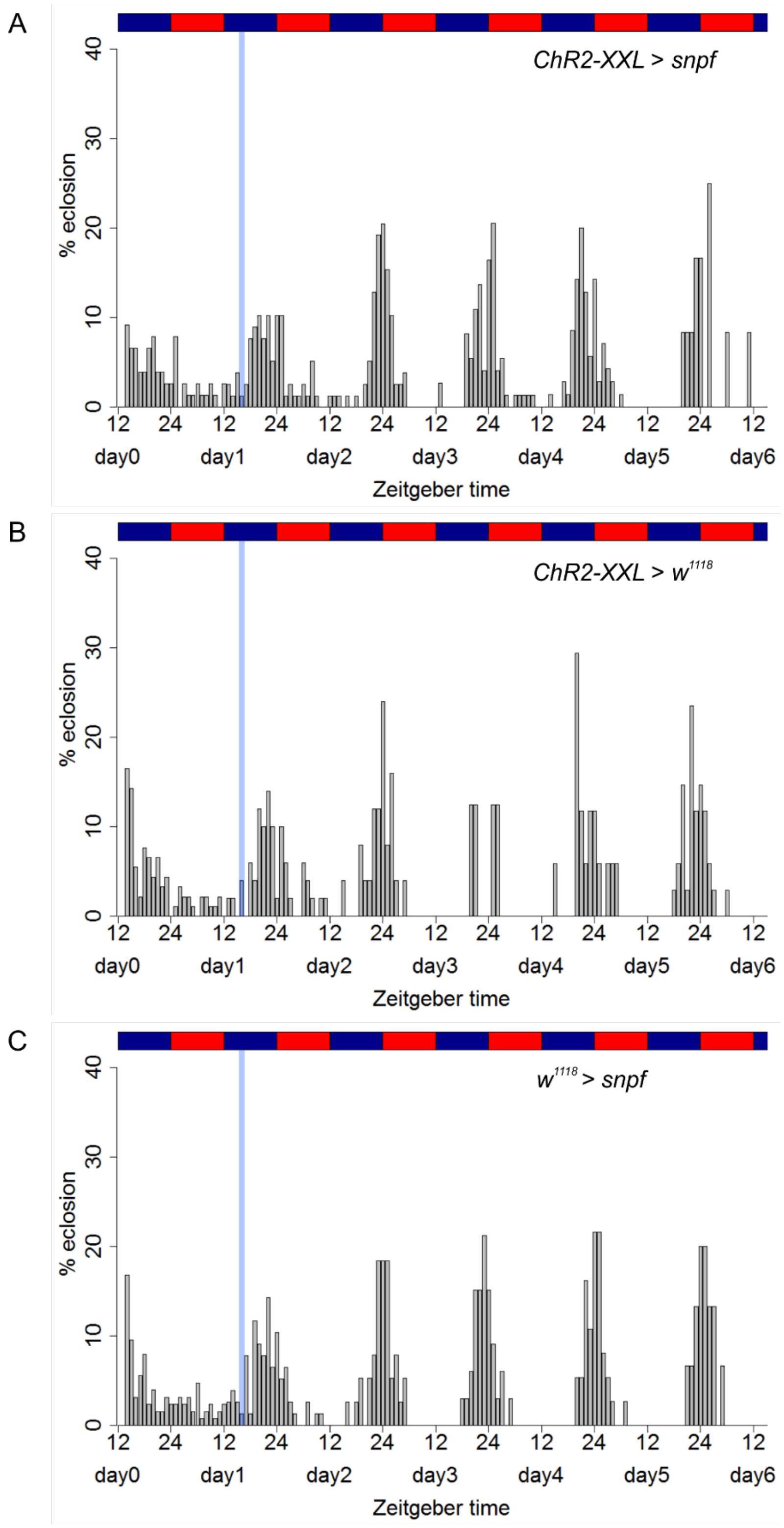

Eclosion profiles with optogenetic activation of *sNPF-Gal4* neurons 6h before the circadian eclosion peak

Eclosion profiles for populations expressing ChR2-XXL driven by *sNPF*-GAL4 (A) and the respective controls (B: UAS control, C: GAL4 control) under temperature entrainment (25°C:16°C). Each bar represents the percentage of eclosed flies per hour normalized to the number of eclosed flies per day. The blue and red rectangles represent the temperature regime. The blue bars mark the time points of activation with blue light. (N=3, 3, 3; n=387, 225, 325)

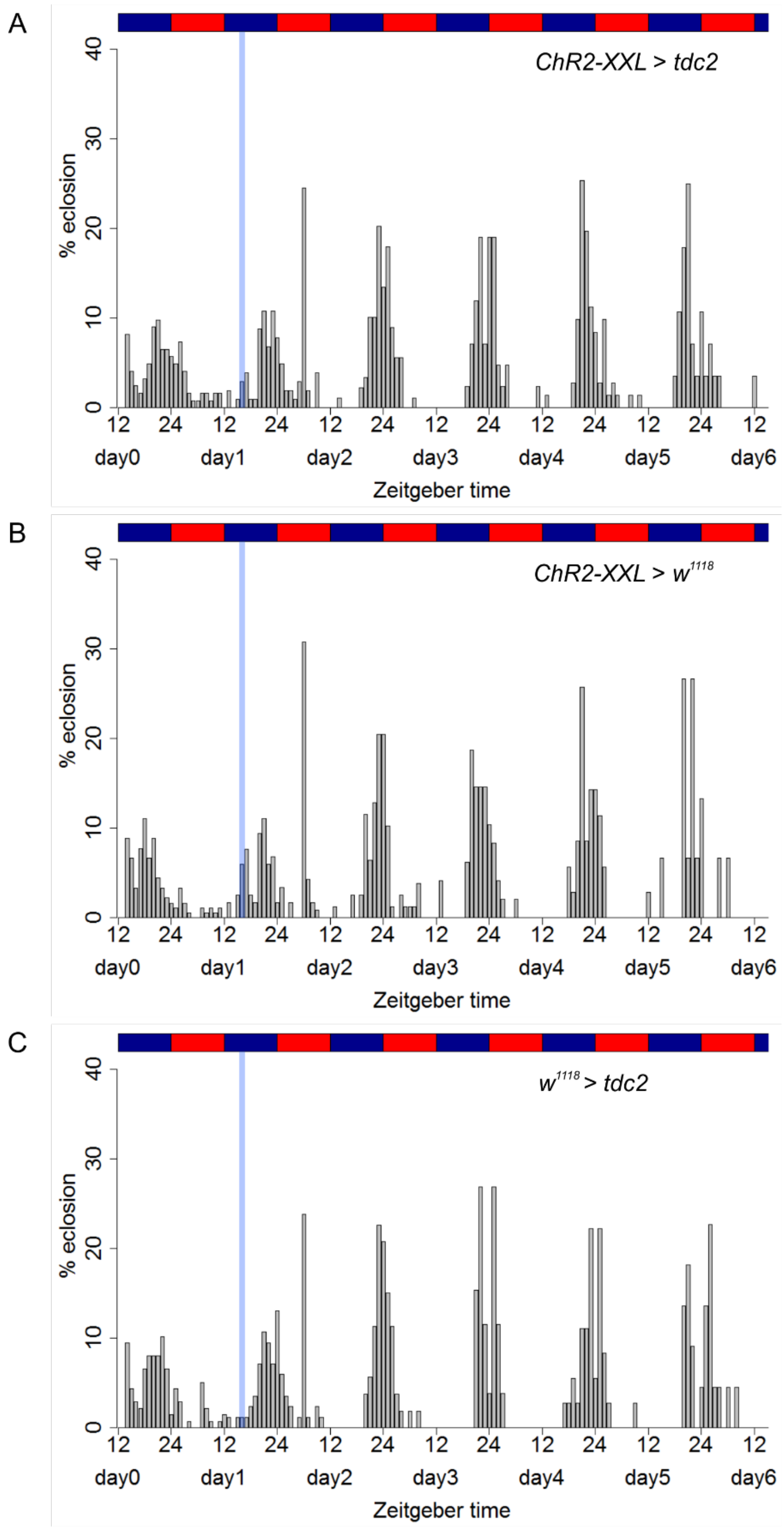

Eclosion profiles with optogenetic activation of *Tdc2-Gal4* neurons 6h before the circadian eclosion peak

Eclosion profiles for populations expressing ChR2-XXL driven by *Tdc2*-GAL4 (A) and the respective controls (B: UAS control, C: GAL4 control) under temperature entrainment (25°C:16°C). Each bar represents the percentage of eclosed flies per hour normalized to the number of eclosed flies per day. The blue and red rectangles represent the temperature regime. The blue bars mark the time points of activation with blue light. (N=3, 3, 3; n=465, 478, 360)

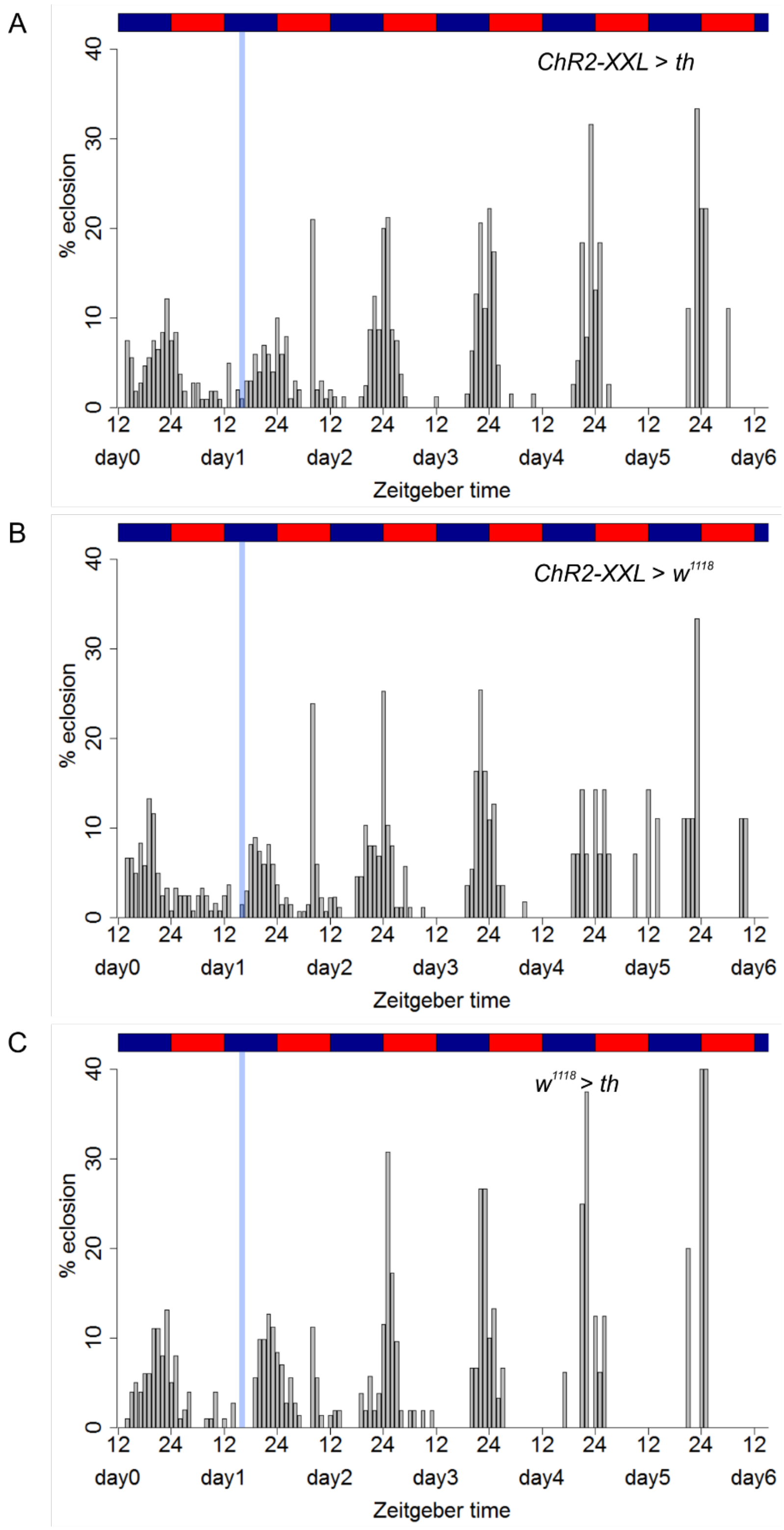

Eclosion profiles with optogenetic activation of *Th-Gal4* neurons 6h before the circadian eclosion peak

Eclosion profiles for populations expressing ChR2-XXL driven by *Th*-GAL4 (A) and the respective controls (B: UAS control, C: GAL4 control) under temperature entrainment (25°C:16°C). Each bar represents the percentage of eclosed flies per hour normalized to the number of eclosed flies per day. The blue and red rectangles represent the temperature regime. The blue bars mark the time points of activation with blue light. (N=3, 3, 3; n=402, 428, 277)

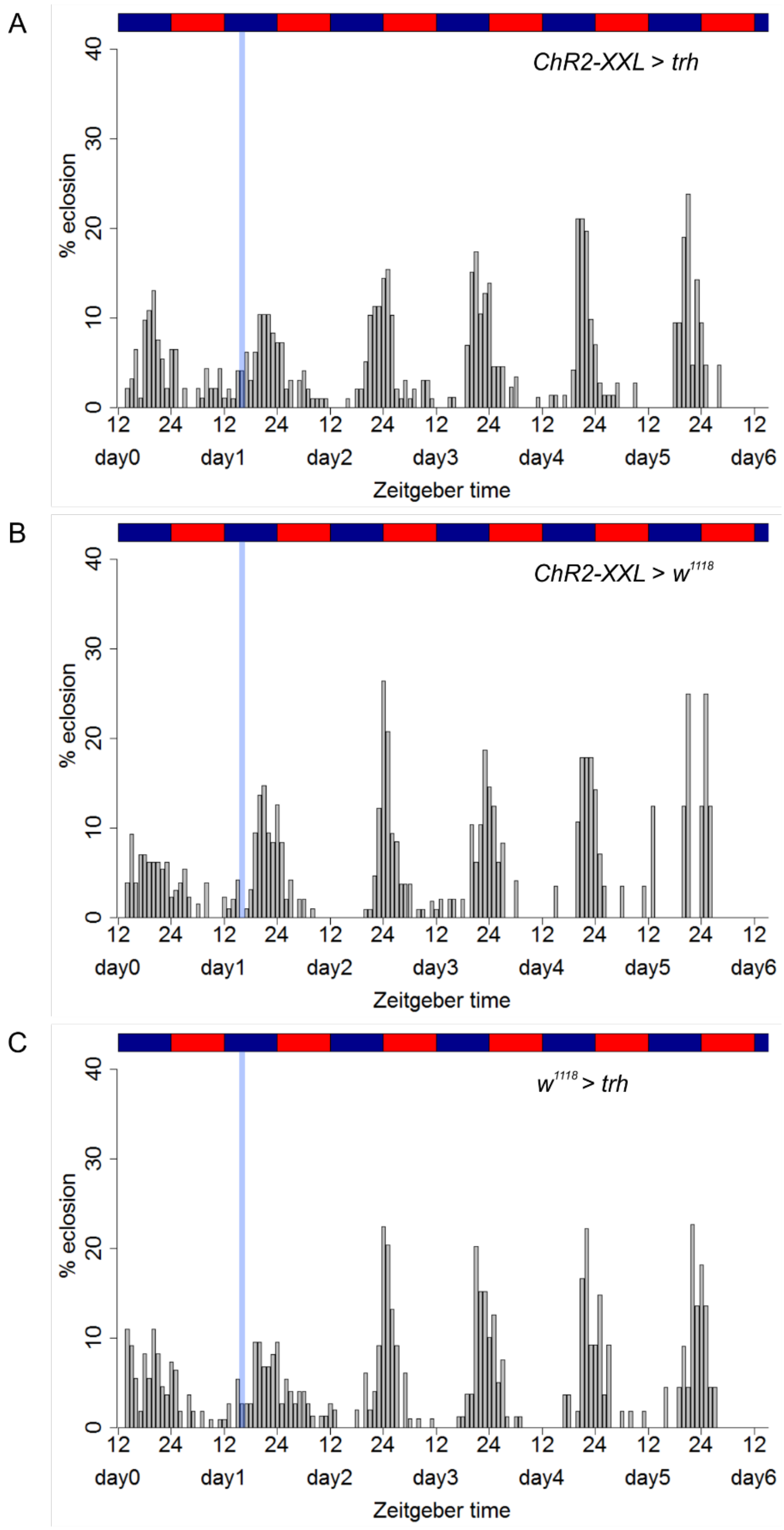

Eclosion profiles with optogenetic activation of *Trh-Gal4* neurons 6h before the circadian eclosion peak

Eclosion profiles for populations expressing ChR2-XXL driven by *Trh*-GAL4 (A) and the respective controls (B: UAS control, C: GAL4 control) under temperature entrainment (25°C:16°C). Each bar represents the percentage of eclosed flies per hour normalized to Discussion
