# Supplementary material for: WEclMon – A simple and robust camera-based system to monitor Drosophila eclosion under optogenetic manipulation and natural conditions
Source: PLoS One. 2017 Jun 28;12(6):e0180238. doi: 10.1371/journal.pone.0180238 (PMC5489222; doi:10.1371/journal.pone.0180238)
Supplement: S2 Fig — (PDF) [file pone.0180238.s005.pdf]

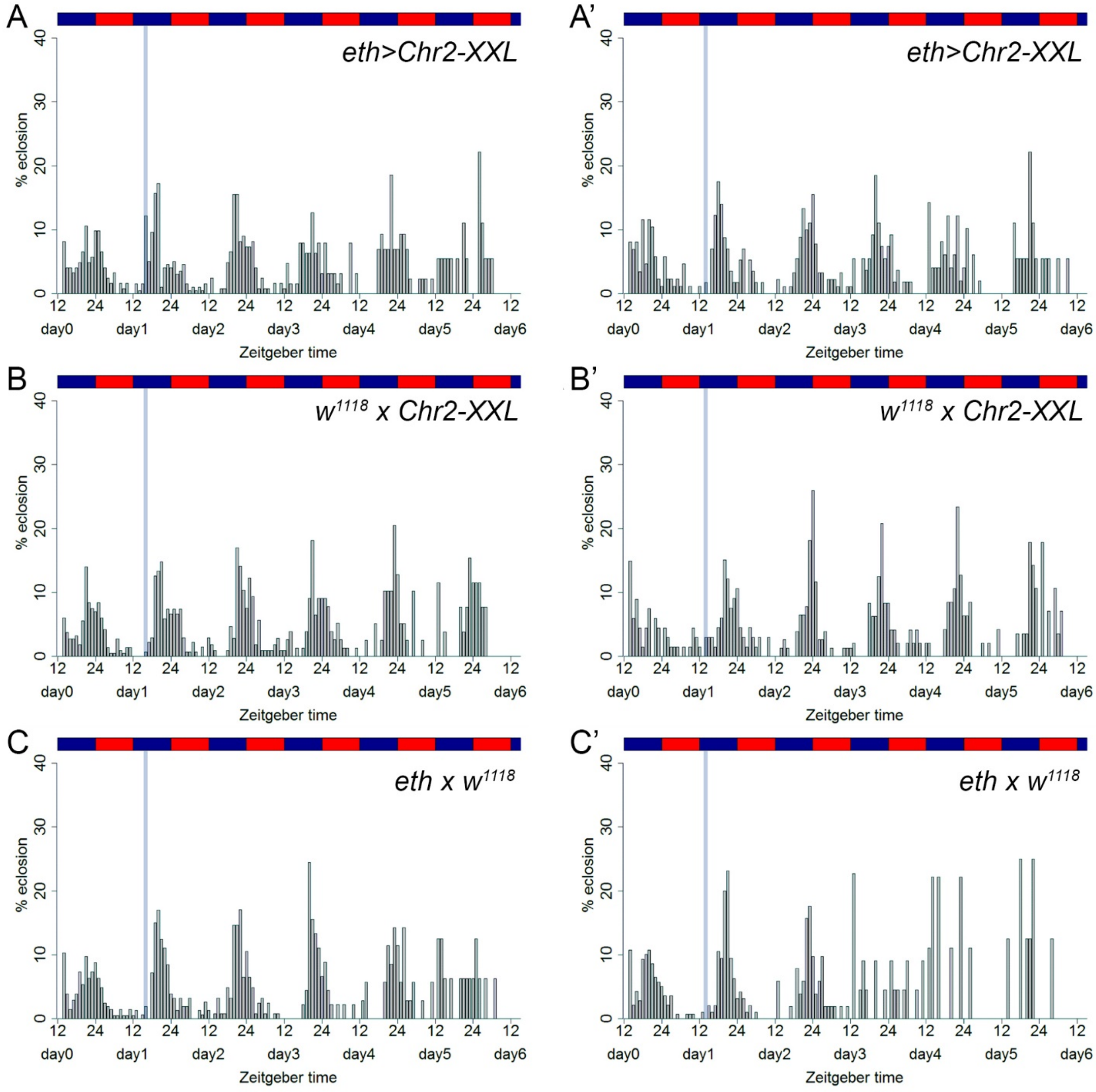

S2 Fig: Eclosion profiles with optogenetic activation of ETH-Gal4 neurons 6h and 8h before the circadian eclosion peak. A 1h blue light pulse was given either 6h (A-C) or 8h (A'-C') before the eclosion peak in flies expressing Chr2-XXL in the epitracheal cells (A, A'), or UAS- (B, B') and Gal4 –controls (C, C'). A precocious eclosion peak could only be induced by activating epitracheal cells 6h before (A), but not 8h before the circadia eclosion peak (A'). Optogenetic activation had no effect in genetic controls independent of the timing of the blue light pulse (B-C').A 1h blue light pulse was given either 6h (A-C) or 8h (A'-C') before the eclosion peak in flies expressing Chr2-XXL in the epitracheal cells (A, A'), or UAS- (B, B') and Gal4 –controls (C, C'). A precocious eclosion peak could only be induced in flies with activatable epitracheal cells 6h before (A), but not 8h before the eclosion peak (A'). Optogenetic activation had no effect in genetic controls independent of the timing of the blue light pulse (B-C').
